# GM-CSF-Producing Th Cells in Rats Sensitive and Resistant to Experimental Autoimmune Encephalomyelitis

**DOI:** 10.1371/journal.pone.0166498

**Published:** 2016-11-10

**Authors:** Zorica Stojić-Vukanić, Ivan Pilipović, Ivana Vujnović, Mirjana Nacka-Aleksić, Raisa Petrović, Nevena Arsenović-Ranin, Mirjana Dimitrijević, Gordana Leposavić

**Affiliations:** 1 Department of Microbiology and Immunology, University of Belgrade-Faculty of Pharmacy, Belgrade, Serbia; 2 Immunology Research Center “Branislav Janković”, Institute of Virology, Vaccines and Sera “Torlak”, Belgrade, Serbia; 3 Department of Physiology, University of Belgrade-Faculty of Pharmacy, Belgrade, Serbia; 4 Department of Immunology, Institute for Biological Research "Siniša Stanković", University of Belgrade, Belgrade, Serbia; Queen Mary University of London, UNITED KINGDOM

## Abstract

Given that granulocyte macrophage colony-stimulating factor (GM-CSF) is identified as the key factor to endow auto-reactive Th cells with the potential to induce neuroinflammation in experimental autoimmune encephalomyelitis (EAE) models, the frequency and phenotype of GM-CSF-producing (GM-CSF+) Th cells in draining lymph nodes (dLNs) and spinal cord (SC) of Albino Oxford (AO) and Dark Agouti (DA) rats immunized for EAE were examined. The generation of neuroantigen-specific GM-CSF+ Th lymphocytes was impaired in dLNs of AO rats (relatively resistant to EAE induction) compared with their DA counterparts (susceptible to EAE) reflecting impaired CD4+ lymphocyte proliferation and less supportive of GM-CSF+ Th cell differentiation dLN cytokine microenvironment. Immunophenotyping of GM-CSF+ Th cells showed their phenotypic heterogeneity in both strains and revealed lower frequency of IL-17+IFN-γ+, IL-17+IFN-γ-, and IL-17-IFN-γ+ cells accompanied by higher frequency of IL-17-IFN-γ- cells among them in AO than in DA rats. Compared with DA, in AO rats was also found (i) slightly lower surface density of CCR2 (drives accumulation of highly pathogenic GM-CSF+IFN-γ+ Th17 cells in SC) on GM-CSF+IFN-γ+ Th17 lymphocytes from dLNs, and (ii) diminished CCL2 mRNA expression in SC tissue, suggesting their impaired migration into the SC. Moreover, dLN and SC cytokine environments in AO rats were shown to be less supportive of GM-CSF+IFN-γ+ Th17 cell differentiation (judging by lower expression of mRNAs for IL-1β, IL-6 and IL-23/p19). In accordance with the (i) lower frequency of GM-CSF+ Th cells in dLNs and SC of AO rats and their lower GM-CSF production, and (ii) impaired CCL2 expression in the SC tissue, the proportion of proinflammatory monocytes among peripheral blood cells and their progeny (CD45^hi^ cells) among the SC CD11b+ cells were reduced in AO compared with DA rats. Collectively, the results indicate that the strain specificities in efficacy of several mechanisms controlling (auto)reactive CD4+ lymphocyte expansion/differentiation into the cells with pathogenic phenotype and migration of the latter to the SC contribute to AO rat resistance to EAE.

## Introduction

Experimental autoimmune encephalomyelitis (EAE) is the most frequently used model system for studying multiple sclerosis (MS) in laboratory animals. Rather than a single model, EAE is a family of models in which central nervous system (CNS) inflammation occurs after immunization of susceptible animal strains with nervous tissue or myelin peptides, such as myelin basic protein (MBP) and proteolipid protein in adjuvant [1]. The specific pathological and clinical features vary dramatically dependent on the animal species, genetic (sub)strain, induction protocol, and autoantigen used, replicating different aspects of MS [2–6]. Depending on the model, EAE may develop in highly distinct forms such as acute, relapsing-remitting, and primary or even secondary progressive [7]. It has been clearly proven that CD4+ T lymphocytes are the major driver of the disease when rodents are immunized by CNS antigen(s) in complete Freund's adjuvant (CFA) [8]. Consequently, this EAE model is used as a prototype for CD4+ T lymphocyte-mediated autoimmune diseases [9]. Although Th1 and Th17 cells have been implicated in development of EAE [10–12], an accumulating body of evidence indicates that neither IFN-γ nor IL-17 (Th1 and Th17 signature cytokines, respectively) is indispensable in its pathogenesis [13–16]. Furthermore, active immunization of donor Csf2^–/–^mice elicited neuroantigen specific T cells that secreted IFN-γ and IL-17, but these T cells did not mediate the adoptive transfer of EAE, suggesting that they are not sufficient to secure EAE development [17,18]. However, adoptive transfer of granulocyte macrophage colony-stimulating factor (GM-CSF) sufficient effector T cells that were deficient in both IFN-γ and IL-17 caused severe EAE commensurate with wild type T cells. Based on these observations, GM-CSF is widely considered to be the signature cytokine of pathogenic effector T cells in EAE, and therefore one of the few cytokines critical for EAE [17–20]. Consequently, Th lymphocyte-derived GM-CSF was suggested to be of pivotal importance for susceptibility of distinct mouse strains to EAE [18]. Additionally, GM-CSF has attracted substantial attention as a result of the potential for antibody-mediated clinical intervention [21]. Considering all the aforementioned, it is understandable that driving factors and mechanisms underlying differentiation of GM-CSF-producing (GM-CSF+) Th lymphocytes and its role in the development of autoimmune diseases are gaining increasing attention.

All conventional Th cell subsets in mice and humans produce GM-CSF [19,22]. However, IL-7/STAT5 signalling axis-induced CD4+ T lymphocytes are shown to be the main source of GM-CSF in autoimmune neuroinflammation in mice [19]. They most probably represent a new Th lymphocyte subset [19]. This subset characterized by a distinct differentiation program and cytokine production profile (as it does not produce Th1, Th2, Th17 signature cytokines) is designated as Th-GM subset [19]. A similar Th cell subset, but with distinct developmental requirements, has also been identified in humans [22]. GM-CSF is shown to accelerate the release of bone marrow precursors and their recruitment into the CNS parenchyma, where they differentiate into inflammatory macrophages and dendritic cells [23]. These cells, in turn, promote: (i) re-activation/differentiation of GM-CSF+ Th lymphocytes infiltrating the CNS and (ii) nervous tissue destruction through release of various damaging molecules [24–26]. The contribution of distinct GM-CSF+ Th cell subsets (Th17, Th1, Th-GM) to autoimmune neuroinflammation has yet not been defined. However, it has been shown that the pathogenicity of autoreactive Th17 cells in mice and, possibly in rats is linked with their production of GM-CSF [17,18,27]. Given that Th17 lymphocyte response is promoted in CFA-induced models of autoimmunity [12], and that more severe EAE in the rat is associated with higher frequency of Th17 cells co-producing IFN-γ and IL-17 in spinal cord (SC) [27,28], multi-cytokine producing Th17 lymphocytes are worthy of special attention in studies of rat susceptibility to EAE.

Differentiation of Th cell subsets is accompanied by sequential expression of selectins, integrins, and chemokine receptors responsible for their recruitment to and extravasation at inflammation sites [29]. Consistently, their expression has a significant impact on development of autoimmune diseases, including MS and EAE [30–32]. It has been suggested that the chemokine CCL20 and its receptor CCR6, a common marker of Th17 cells [33] and certain tissue-homing T regulatory (Treg) cells [34,35] are involved in pathogenesis of MS and EAE [31,32,36]. Recently published data indicate that CCR2, but not CCR6, is the key driver of encephalitogenic Th17 cell recruitment into the CNS [37]. Furthermore, in mouse EAE model, highly pathogenic GM-CSF/IFN-γ-producing Th17 cells are identified as cells bearing a CCR6^−^CCR2^+^ phenotype [37].

Having all the aforementioned in mind, as well as that there is no data on the role of GM-CSF+ Th cells in rat strains sensitive and resistant to EAE induction, the study was designed to assess the putative differences in draining lymph node (dLN) generation of GM-CSF+ Th cells, particularly Th17 cells co-producing IFN-γ and GM-CSF, and their infiltration into SC between relatively resistant to EAE induction Albino Oxford (AO) and susceptible to the disease Dark Agouti (DA) rats [38–40]. We chose AO rats considering that the increase in their sensitivity to EAE induction with aging coincides with the increase in encephalitogenic GM-CSF+ Th-cell generation in dLNs/SC and higher frequency of GM-CSF+ Th cells infiltrating the SC [28].

## Materials and Methods

### Experimental animals

Female young (4-5-month-old) AO (192±3.7 g) and DA (155±5.8 g) rats from a breeding colony of the Immunology Research Center “Branislav Janković” in Belgrade were used in the present study. The animal facilities were accredited by Ministry of Agriculture and Enviromental Protection of the Republic of Serbia (Veterinary Department). Animals were bred and housed (3 rats/polyethylene cage containing sterilized wood shavings, under controlled humidity, temperature and lighting conditions) according to EU directive 2010/63/EU and the governmental regulations (Law on Animal Welfare, “Official Gazette of RS”, no. 14/2009). All animals received routine care, including feeding standard diets, providing fresh water *ad libitum*, and changing cages and bedding. Animal health monitoring was performed on a daily basis by animal care staff and a veterinarian.

### Induction and clinical evaluation of EAE

To induce active EAE, anesthetized rats (48 in each of two independent experiments) received injections of 100 μl of an emulsion made of equal volumes of rat SC homogenate in phosphate-buffered saline (PBS) and CFA containing 1 mg/ml of heat-killed and dried *Mycobacterium tuberculosis* H37Ra (Sigma-Aldrich Chemie GmbH, Taufkirchen, Germany) into the left hind foot pad, and 0.25 ml of saline suspension of 5x10^8^ *Bordetella pertussis* (Institute of Virology, Vaccines and Sera "Torlak", Belgrade, Serbia) subcutaneously into the dorsum of the same paw [27,41]. To minimize stress, pain and injury, rats were anesthetized with an intraperitoneal injection of ketamine (Ketamidor, Richter Pharma AG, Wels, Austria; 100 mg/ml)/xylazine (Xylased, Bioveta, Ivanovice na Hané, Czech Republic; 20 mg/ml) anesthetizing cocktail [50 mg/kg body weight (BW) of ketamine/5 mg/kg BW xylazine]. In DA rats, this immunization protocol induces acute monophasic disease followed by full recovery of all animals [27,41]. Consistently, none of DA rats died at any time before experimental endpoints. Immunized rats were monitored starting at the 1^st^ day post-immunization (d.p.i.) twice daily. Neurological scores (0, no clinical signs; 0.5, distal tail atony; 1, complete tail atony; 2, paraparesis; 3, paraplegia; 4, tetraplegia or moribund state) and animal weight were recorded daily by two independent experienced observers. The activity of animals was monitored over 5 minutes. None of the rats reached moribundity during the studies. For animals that developed neurological signs of EAE mashed food and water were positioned lower to facilitate access to food and hydration and thereby to improve welfare assistance and clinical status. Only one DA rat progressed to tetraplegia (score 4) on the 13^th^ d.p.i. None of rats experienced reduction in body weight greater than 15%. Animals were sacrificed either in the inductive phase of EAE, on the 7^th^ d.p.i. or on the 13^th^ d.p.i., when the disease in DA rats reaches peak [27,41], through transcardial perfusion. Prior to transcardial perfusion the animals were deeply anesthetized with an intraperitoneal injection of ketamine/xylazine anesthetizing cocktail (80 mg/kg BW/8 mg/kg BW). All animals were handled and treated in complete compliance with the Directive 2010/63/EU of the European Parliament and of the Council on the protection of animals used for scientific purposes and Institutional guidelines were approved by the Animal Care and Use Committee of the Faculty of Pharmacy (permit number 6/12).

### Antibodies and immunoconjugates

Monoclonal antibodies (mAbs) to rat CD4, CD8, CD134, CD45, CD11b, TCRαβ, IFN-γ, IL-17A, RT1B (MHC II), CD40, CD45RA, CD62L, CD32, IL-4, secondary reagents and isotype controls were obtained from BD Biosciences Pharmingen (Mountain View, CA, USA). Additional mAbs to rat CD11b (Serotec, Oxford, UK), CD25 and IL-17A (eBioscience, San Diego, CA, USA), TCRαβ and CD43 (BioLegend, San Diego, CA, USA), CCR2 and CCR6 (R&D Systems, Inc., Minneapolis, MN, USA), and GM-CSF (Novus Biologicals, Littleton, CO, USA) were also used. Polyclonal Abs to CX3CR1 and CCR7 were acquired from Abcam (Cambridge, UK).

### Isolation of mononuclear cells

Following transcardial perfusion with PBS, rat SCs and dLNs were carefully removed, grinded on 70 μm nylon cell strainer (BD Biosciences, Erembodegem, Belgium) to retrieve mononuclear cells. Thereby obtained mononuclear cells were collected in Petri dishes containing either PBS supplemented with 2% fetal calf serum (FCS, Gibco, Grand Island, NY, USA) and 0.01% NaN_3_ (Sigma-Aldrich Chemie GmbH) (FACS buffer) or RPMI 1640 medium (Sigma-Aldrich Chemie GmbH) supplemented with 5% FCS (SC mononuclear cells). SC cells were fractioned on a discontinuous 40/70% Percoll (Sigma-Aldrich Chemie GmbH) gradient at 1000x*g* for 50 min and mononuclear cells from the interphase were collected. Blood samples were taken (on the 7^th^ d.p.i.) prior to the perfusion by cardiac puncture and subjected to NH_4_Cl lyses to remove red blood cells. Thereby obtained blood cells, as well as dLN cells and mononuclear SC cells, were counted in 0.2% trypan blue solution using an improved Neubauer hemacytometer.

### Separation of CD4+ T cells

To purify CD4+ T cells from mononuclear SC and dLN cell suspensions a two-step magnetic-activated cell sorting (MACS) procedure, using the equipment and reagents supplied by Miltenyi Biotec (Gladbach, Germany), was applied [28]. The cells were labeled using rat CD8a microbeads and loaded onto LS column, placed in the magnetic field of the Quadro MACS separator, for negative selection. Thereby obtained CD8- cell fraction was incubated with rat pan T-cell microbeads and positively selected for T lymphocytes. The positive fractions, containing 90–95% of CD4+ T cells, as shown by flow cytometry analysis (FCA), were collected for further analyses.

### Stimulation of SC and dLN mononuclear cells for analyses of cytokine production

Purified CD4+ T cells from SCs and dLNs were cultured in complete RPMI 1640 culture medium supplemented with 200 ng/ml phorbol 12-myristate 13-acetate (PMA, Sigma-Aldrich Chemie GmbH) and 400 ng/ml ionomycine (Sigma-Aldrich Chemie GmbH) in a 5% CO_2_ humidified atmosphere for 4 h at 37°C. For intracellular staining of cytokines 3 μg/ml of brefeldin A (eBioscience) was added 2 h before the end of the assay. From SC cell cultures without brefeldin A supernatants were collected for cytokine ELISA.

### Cultivation of dLN cells for analyses of cytokine production and cell proliferation

Cells from dLNs were cultured for 72 h without or with 2.5 μg/ml of Concanavalin A (ConA, Sigma-Aldrich Chemie GmbH) or 20 μg/ml of MBP (Sigma-Aldrich Chemie GmbH) in a 5% CO_2_ humidified air atmosphere at 37°C. Supernatants were harvested and assayed for IL-17 and GM-CSF by ELISA, while the cells were processed for cell cycle analysis or restimulated with PMA and ionomycine for intracellular cytokine immunostaining.

### RT-qPCR

Spinal cord and dLN cell and tissue samples were collected using Nucleic Acid Purification Lysis Solution (Applied Biosystems, Foster City, CA, USA) and immediately stored at -70°C until RNA purification. Total RNA from cell and tissue samples was extracted using ABI Prism 6100 Nucleic Acid PrepStation system (Applied Biosystems) and Total RNA Chemistry Starter Kit (Applied Biosystems), including DNAse (Absolute RNA Wash Solution, Applied Biosystems) treatment to ensure that no genomic DNA contamination was present. cDNA was synthesized using High Capacity cDNA Reverse Transcription Kit (Applied Biosystems), in 20-μl reactions with the thermal cycler conditions set as follows: 10 min at 25°C, 120 min at 37°C and 5 sec at 85°C.

RT-qPCR reaction mixtures contained 5 μl of cDNA template, 1x TaqMan Gene Expression Master Mix with Uracil-DNA glycosylase (UDG) (Applied Biosystems) and 1x mix of premade primer and hydrolysis probe sets (TaqMan Gene Expression Assays, Applied Biosystems) in a total volume of 25 μl. The triplicate RT-qPCR reactions were performed using Applied Biosystems 7500 Real-Time PCR System under pre-optimized conditions: 2 min at 50°C (UDG incubation), 10 min at 95°C (AmpliTaq Gold DNA Polymerase activation), and 40 cycles including 15 sec at 95°C (template denaturation) and 1 min at 60°C (primer annealing/extension).

All the procedures were performed according to the manufacturer’s instructions. TaqMan Gene Expression Assays used in the study are listed in S1 Table. Target mRNA levels were quantified by the 2^-ΔΔCt^ method with SDS v1.4.0. software (Applied Biosystems), using β-actin as a normalizer, as it has been previously suggested [28,41].

### FCA

For immunostaining, all incubation steps were performed at 4°C, unless stated otherwise. Samples were acquired on FACSCalibur or FACSVerse flow cytometer (Becton Dickinson, Mountain View, CA, USA). The data were analyzed for percentage of marker positive cells and/or mean fluorescence intensity (MFI) using FlowJo software version 7.8. (TreeStar Inc, Ashland, OR, USA). IgG isotype-matched controls were used for each fluorochrome type and fluorescence minus one controls were applied to settle gating boundaries, as previously shown [41].

#### Surface antigen immunostaining

Cells were incubated with saturating concentrations of either fluorochrome-labeled mAbs or biotin-conjugated/unconjugated Abs for 30 min and washed in FACS buffer. When biotin-conjugated/unconjugated Abs were applied, cells were incubated with appropriate second-step reagents for additional 30 min, washed and collected for FCA.

#### Intracellular antigen immunostaining

Following stimulation with PMA and ionomycine, MACS-separated SC and dLN CD4+ T cells and surface-stained SC and cultivated dLN mononuclear cells were fixed/permeabilized overnight at 4°C, using the fixation/permeabilization buffer kit (eBioscience), according to the manufacturer’s instructions (eBioscience; http://www.ebioscience.com/resources/best-protocols/flow-cytometry-protocols.htm). For intracellular cytokine content assessment, washed cells were stained with fluorochrome-conjugated mAbs to IL-17, IFN-γ and GM-CSF for 30 min at room temperature in the dark, washed again and collected for FCA.

For detection of CD4+CD25+ Treg lymphocytes, dLN cells were surface-stained for CD4 and CD25, fixed/permeabilized using the reagents from the Foxp3 Staining Set (eBioscience), and stained with FITC-conjugated anti-FoxP3 mAb, as suggested by the manufacturer.

### CD4+ dLN cell proliferation *in vitro*

The proliferating cells among CD4+ dLN lymphocytes were identified combining CD4/CD8 surface antigen labeling with 7-AAD DNA staining. Briefly, cultured dLN cells were incubated with a cocktail of fluorochrome-conjugated anti-CD4 and anti-CD8 mAbs for 30 min in the dark at 4°C, and then washed in cold PBS. The pellet was resuspended in 150 μl of 50% FCS in PBS, and the cells were fixed/permeabilized by 450 μl of cold 70% ethanol in double distilled H_2_O. Next, the cells were washed twice with cold PBS to remove the ethanol and the precipitated proteins and incubated with 7-AAD (BD Pharmingen, 10 μl) for 30 min at 4°C. Doublets were excluded by analyzing the correlated area against the width signals of 7-AAD fluorescence on doublet discrimination module (DDM) dot plot. The frequency of proliferating cells was determined using Dean-Jet-Fox model of the cell cycle platform generated by FlowJo software version 7.8. (TreeStar Inc, Ashland, OR, USA).

### ELISA

Commercial ELISA kits were used for measuring IL-17 (BioLegend; 8 pg/ml detection limit) and GM-CSF (Elabscience Biotechnology Co., Ltd, Wuhan, China; 9.375 pg/ml detection limit) concentrations.

### Statistical analysis

Group mean comparisons were performed with GraphPad Prism 5 software (GraphPad Software, Inc., La Jolla, CA, USA), using unpaired Student's *t*-test or One-way ANOVA followed by Tukey’s post-test. Values of *p*≤0.05 were considered significant.

## Results

As previously shown [28,39], although minimal single cell infiltrate is regularly seen (independently on immunization protocol) in SC of young AO rats [39], they did not develop neurological signs of EAE. On the contrary, DA rats developed clinically manifested disease. As expected [41], the disease in DA rat exhibited monophasic paralytical course with the peak on the 13^th^ d.p.i. and spontaneous recovery (S1 Fig).

### Strain differences in phenotypic characteristics of CD4+ dLN T lymphocytes isolated on the 7^th^ d.p.i. from rats immunized for EAE

#### Strain differences in the number of activated rat CD4+ dLN T lymphocytes

**Fig 1:**
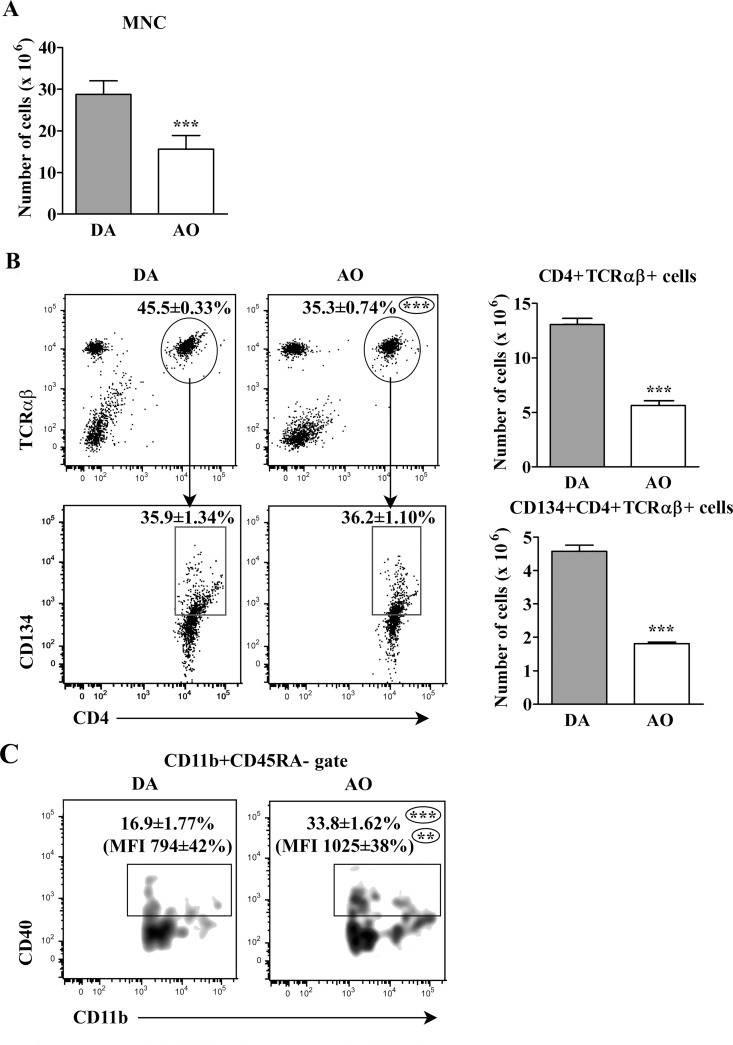
Fewer activated CD4+TCRαβ+ cells in draining lymph nodes of AO rats immunizated for EAE compared with their DA counterparts. **(A)** Bar graph indicates the number of mononuclear cells (MNC) retrieved from draining lymph nodes (dLNs) of DA and AO rats on the 7^th^ d.p.i. **(B)** Flow cytometry dot plots show the frequency of (lower) CD134+ cells within (upper) CD4+TCRαβ+ lymphocytes from dLNs of DA and AO rats on the 7^th^ d.p.i. The bar graphs indicate number of (upper) CD4+TCRαβ+ and (lower) CD134+CD4+TCRαβ+ lymphocytes in dLNs from DA and AO rats on the 7^th^ d.p.i. **(C)** Flow cytometry density plots show the frequency of CD11b+CD40+ cells within CD11b+CD45RA- cells (gated as depicted in S2 Fig) and CD40 mean fluorescence intensity (MFI) on CD11b+CD40+ cells. Data (mean ± SEM) are representative of two experiments (n = 6). ** p≤0.01; *** p≤0.001.

In accordance with the previous study [40], on the 7^th^ d.p.i. fewer (p≤0.001) mononuclear cells were retrieved from dLNs of AO than DA rats (Fig 1A). Additionally, the frequency of CD4+ TCRαβ+ cells (T lymphocytes) among these cells and their number were lower (p≤0.001) in AO compared with DA rats (Fig 1B). Given that the frequency of activated CD134+ cells among CD4+ T lymphocytes was comparable in AO and DA rats, their number was lower (p≤0.001) in AO rats (Fig 1B).

To explain this disparity, dLN cells were examined for the frequency of MHC II-expressing CD11b+CD45RA- cells (presumably dendritic cells and macrophages). The frequency of MHC II+ cells among CD11b+CD45RA- cells and MHC II surface density was comparable in AO and DA rats, but their number was lower (p≤0.001) in AO than in DA rats (S2 Fig). On the contrary, more (p≤0.001) cells expressing the costimulatory protein CD40 were found among CD11b+CD45RA- cells from AO than DA rats (Fig 1C). Additionally, CD40 density (judging by MFI) was higher (p≤0.01) on CD40+CD11b+CD45RA- cells from AO compared with DA rats (Fig 1C).

**Fig 2:**
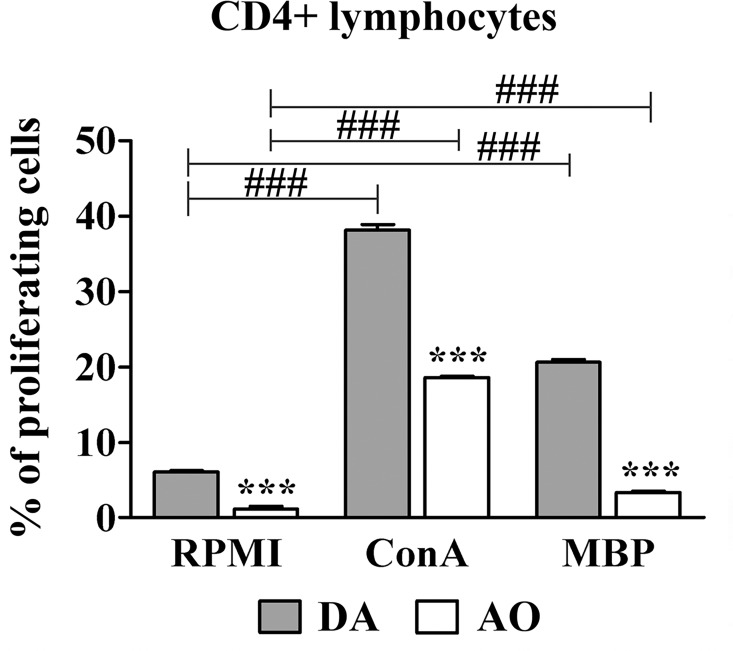
CD4+ draining lymph node cells from AO rats immunized for EAE exhibit lower proliferation in culture than those cells from their DA counterparts. The frequency of proliferating cells (cells in S+G2/M phases of cell cycle) within CD4+ draining lymph node (dLN) lymphocytes retrieved on the 7^th^ d.p.i. from AO and DA rats and cultivated in RPMI alone or in RPMI supplemented with ConA or MBP. The flow cytometry profiles depicting 7-ADD staining of CD4+ dLN lymphocytes are shown in S3 Fig. Data (mean ± SEM) are representative of two experiments (n = 6). *** p≤0.001; ### p≤0.001. * vs DA rats; # vs RPMI.

In both basal (RPMI cultures) and ConA- or MBP-supplemented (ConA or MBP cultures) dLN cell cultures, the frequency of proliferating cells (cells in S+G2/M phases of cell cycle) was lower (p≤0.001) among CD4+ lymphocytes from AO when compared with those cells from DA rats (Fig 2, S3 Fig).

The frequency of CD25+FoxP3+ Treg cells was comparable among CD4+ cells from AO and DA rats (S4 Fig), so the previous findings were not related to differences in their frequency.

#### Strain differences in the frequency and phenotypic characteristics of GM-CSF+ and IL-17+ cells among rat CD4+ dLN T lymphocytes

**Fig 3:**
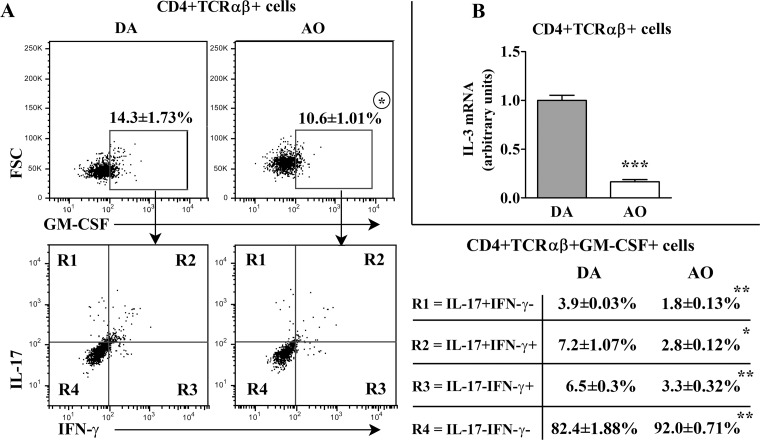
Lower frequency of GM-CSF+ cells within CD4+TCRαβ+ lymphocytes from draining lymph nodes of AO than DA rats immunized for EAE. **(A)** Flow cytometry dot plots show the frequency of (upper) GM-CSF+ cells within CD4+TCRαβ+ draining lymph node (dLN) cells of DA and AO rats on the 7^th^ d.p.i. and (lower) the frequency of their distinct subsets delineated according to IL-17/IFN-γ expression. Table shows the frequency of cells in the indicated region (R). CD4+TCRαβ+ dLN lymphocytes were separated using magnetic-activated cell sorting (MACS) as described in Materials and Methods. The gating strategy is shown in S6 Fig. **(B)** Fold change in IL-3 mRNA expression in CD4+TCRαβ+ dLN cells from AO rats relative to those cells from DA rats. Data (mean ± SEM) are representative of two experiments (n = 6). * p≤0.05; ** p≤0.01; *** p≤0.001.

Next, PMA- and ionomycine-stimulated CD4+ dLN T lymphocytes were examined for the production of GM-CSF. The frequency of GM-CSF+ cells was lower (p≤0.05) within this subpopulation from AO compared with DA rats (Fig 3A). The analysis of GM-CSF+CD4+ T lymphocytes for co-production of IFN-γ and IL-17 also revealed strain differences (Fig 3A). The frequencies of IL-17+IFN-γ+ (p≤0.05), IL-17-IFN-γ+ (p≤0.01) and IL-17+IFN-γ-cells (p≤0.01) were lower in GM-CSF+ CD4+ T-lymphocyte subpopulation from AO rats (Fig 3A). On the contrary, the frequency of IL-17-IFN-γ- cells was higher (p≤0.01) among these cells from AO rats (Fig 3A). Irrespective of the strain, we failed to detect IL-4 production in dLN T lymphocytes (S5 Fig). Thus, it may be assumed that IL-17-IFN-γ- cells belonged to the Th-GM CD4+ T lymphocyte subset [19]. Next, CD4+ T lymphocytes were analyzed for the expression of mRNA for IL-3, the cytokine produced by Th-GM cells in mice [19] and possibly rats [28]. Given that the frequency of all GM-CSF+ cells among CD4+ T lymphocytes was lower in AO rats, despite the higher frequency of IL-17-IFN-γ- cells among them, CD4+ T lymphocytes from AO rats contained less (p≤0.001) amount of IL-3 mRNA than those from DA rats (Fig 3B).

**Fig 4:**
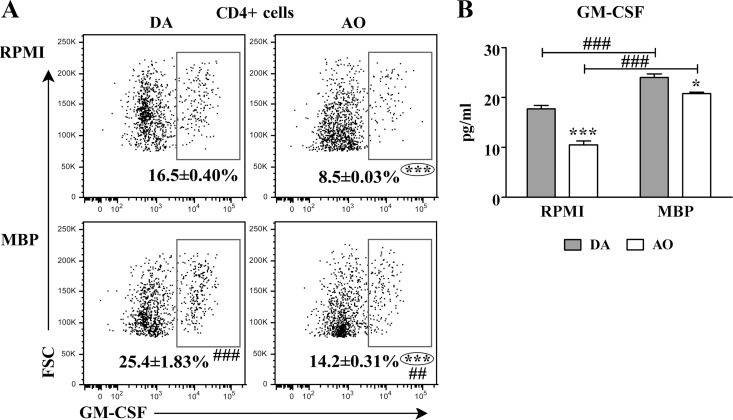
Lower frequency of GM-CSF+ cells within CD4+ lymphocytes from draining lymph node cell cultures of AO than DA rats immunized for EAE. **(A)** Flow cytometry dot plots show the frequency of GM-CSF+ cells within CD4+ draining lymph node (dLN) lymphocytes retrieved from DA and AO rats on the 7^th^ d.p.i. and cultured in RPMI alone or in RPMI supplemented with MBP. Only live cells were gated. **(B)** Concentration of GM-CSF in supernatants of dLN cells from DA and AO rats cultured in RPMI alone or in the presence of MBP. Data (mean ± SEM) are representative of two experiments (n = 6). * p≤0.05; *** p≤0.001; ## p≤0.01; ### p≤0.001. * vs DA rats; # vs RPMI.

In accordance with the previous findings, the frequency of GM-CSF+ cells among CD4+ lymphocytes in both RPMI and MBP cultures from AO rats was lower (p≤0.001) than in the corresponding cultures from DA rats (Fig 4A). Additionally, in MBP cultures from both rat strains the frequency of GM-CSF+ cells within CD4+ lymphocytes was greater (p≤0.01) than in RPMI cultures (Fig 4A). Consistently, the concentration of GM-CSF was lower (p≤0.05) in RPMI and MBP cultures from AO than in the corresponding cultures from DA rats (Fig 4B). It is noteworthy that in rats of both strains GM-CSF concentration was higher (p≤0.001) in MBP than in RPMI cultures (Fig 4B).

**Fig 5:**
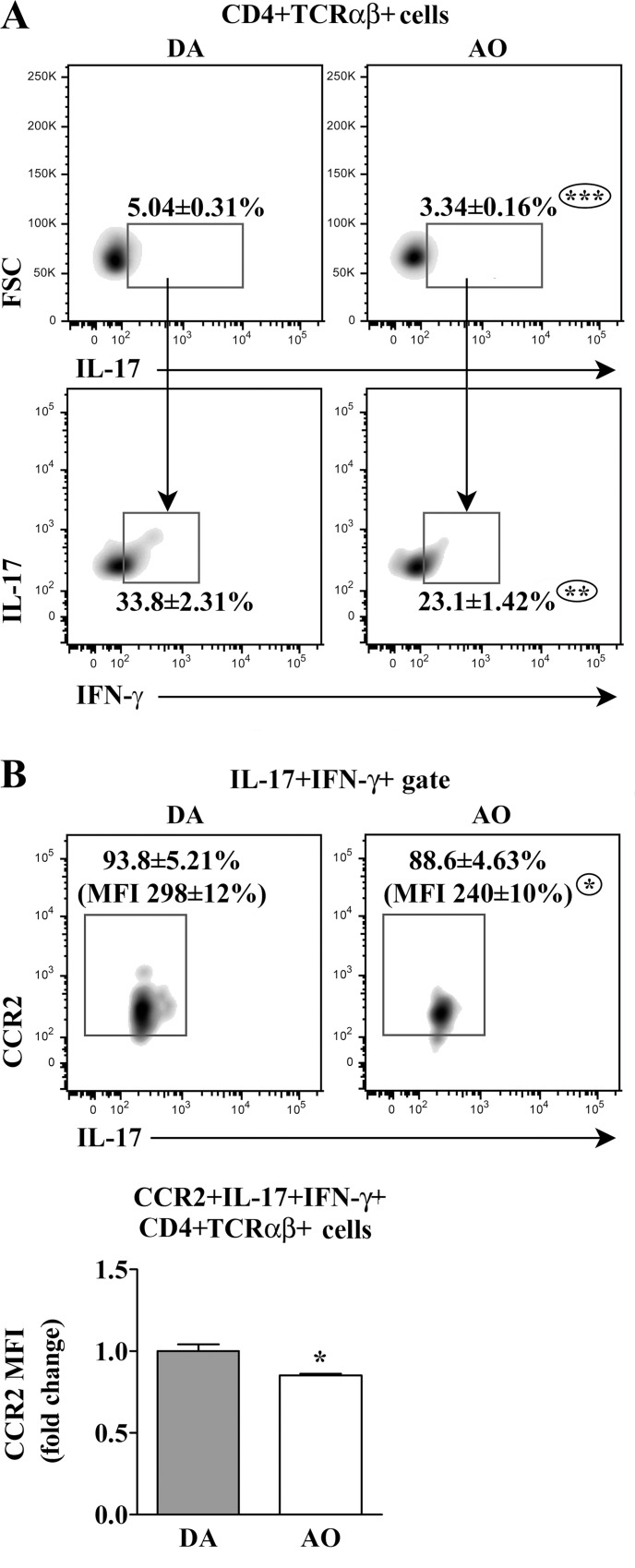
Lower frequency of IL-17+ cells within CD4+TCRαβ+ lymphocytes from draining lymph nodes of AO than DA rats immunized for EAE. **(A)** Flow cytometry density plots show the frequency of (upper) IL-17+ cells within CD4+TCRαβ+ draining lymph node (dLN) lymphocytes retrieved from DA and AO rats on the 7^th^ d.p.i., and (lower) the frequency of IFN-γ-co-producing (IL-17+IFN-γ+) cells within them. **(B)** Flow cytometry density plots indicate the frequency of CCR2+ cells within IL-17+IFN-γ+CD4+TCRαβ+ dLN lymphocytes retrieved from DA and AO rats on the 7^th^ d.p.i. CD4+TCRαβ+ lymphocytes were separated using magnetic-activated cell sorting (MACS) as described in Materials and Methods. The gating strategy is shown in S7 Fig. The numbers in flow cytometry density plots indicate CCR2 mean fluorescence intensity (MFI) on CCR2+ cells. Bar graph shows fold change in CCR2 MFI on CCR2+IL-17+IFN-γ+CD4+TCRαβ+ dLN cells of AO relative to DA rats. Data (mean ± SEM) are representative of two experiments (n = 6). * p≤0.05; ** p≤0.01; *** p≤0.001.

Given that the Th17 response is promoted in CFA-induced models of autoimmunity [12], PMA- and ionomycine-stimulated CD4+ dLN T lymphocytes were also examined for the frequency of IL-17+ cells. Their frequency was lower (p≤0.001) among CD4+ dLN T lymphocytes from AO than DA rats (Fig 5A). Besides, the frequency of IFN-γ co-producing cells was lower (p≤0.01) among IL-17+CD4+ dLN T lymphocytes from AO compared with DA rats (Fig 5A). These cells produce GM-CSF and are highly pathogenic in rodent models of EAE [17,18].

#### Chemokine receptor expression

The migratory properties of effector Th cells are imprinted during differentiation with induction of chemokine receptors that enables their differential trafficking to the inflammatory lesions [37]. Having that in mind, IL-17+ CD4+ T lymphocytes were investigated for the expression of CCR6, a homing receptor shared by Th17 and Tregs [37] and CCR2, a key driver of encephalitogenic IL-17+IFN-γ+ Th cell recruitment into the CNS [37,42]. In both rat strains, almost all IL-17+IFN-γ+ CD4+ T lymphocytes expressed CCR2, but its surface density was slightly lower (p≤0.05) in AO rats (Fig 5B). The frequency of CCR6+ cells among IL-17+ CD4+ T lymphocytes (75±2.23% in DA rats vs 84.32±4.23% in AO rats) and its surface density (judging by MFI) were comparable in AO (384±27) and DA rats (432±22).

**Fig 6:**
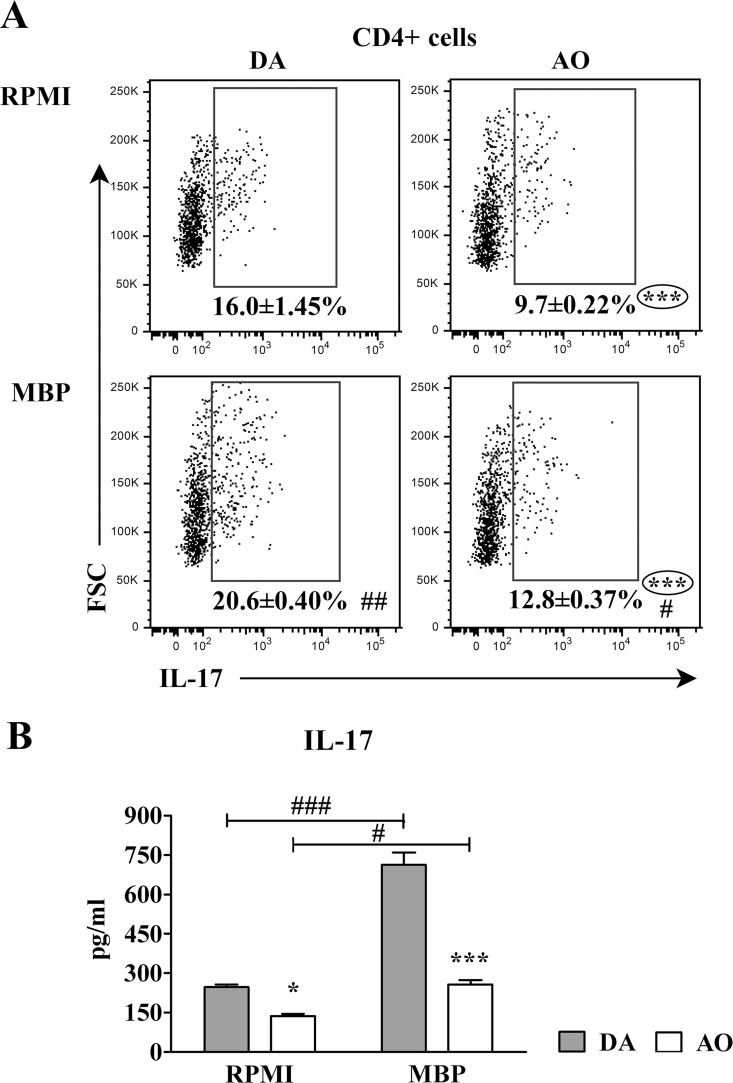
Lower frequency of IL-17+ cells within CD4+ lymphocytes from draining lymph node cell cultures of AO than DA rats immunized for EAE. **(A)** Flow cytometry dot plots show the frequency of IL-17+ cells within CD4+ draining lymph node (dLN) lymphocytes retrieved from DA and AO rats on the 7^th^ d.p.i. and cultured in RPMI alone or in RPMI supplemented with MBP. Only live cells were gated. (**B**) Concentration of IL-17 in supernatants of dLN cells from DA and AO rats cultured in RPMI alone or in the presence of MBP. Data (mean ± SEM) are representative of two experiments (n = 6). * p≤0.05; *** p≤0.001; # p≤0.05; ## p≤0.01; ### p≤0.001. * vs DA rats; # vs RPMI.

In accordance with the previous findings, in both RPMI and MBP cultures from AO rats the frequency of IL-17+ cells among CD4+ lymphocytes was lower (p≤0.001) than in the corresponding cultures from DA rats (Fig 6A). Besides, in MBP cultures from both AO and DA rats the frequency of IL-17+ cells among CD4+ lymphocytes was higher (p≤0.05) than in strain-matched RPMI cultures (Fig 6A). Furthermore, the concentration of IL-17 was lower (p≤0.05) in both RPMI and MBP dLN cell cultures from AO compared with DA rats, but higher (p≤0.05) in MBP than in strain-matched RPMI dLN cell cultures (Fig 6B).

#### Expression of cytokines driving CD4+ T-cell polarization

**Fig 7:**
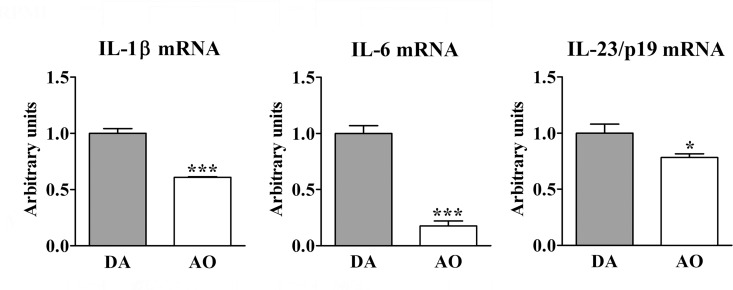
Lower expression of the cytokines driving Th17 cell differentiation in draining lymph node cells from AO compared with DA rats immunized for EAE. Fold change in IL-1β, IL-6 and IL-23/p19 mRNA expression in draining lymph node (dLN) cells from AO rats immunized for EAE relative to those cells from their DA counterparts. Data (mean ± SEM) are representative of two experiments (n = 6). * p≤0.05; *** p≤0.001.

Next, the expression of cytokines driving IL-17 and GM-CSF expression in CD4+ T lymphocytes was investigated. The expression of mRNA for IL-1β (p≤0.001), IL-6 (p≤0.001) and IL-23/p19 (p≤0.05), the cytokines driving differentiation of Th17 cells [43–45], including highly pathogenic multi-cytokine-producing Th17 cells [17,18], was decreased in dLN cells from AO compared with DA rats (Fig 7).

Additionally, despite the higher frequency of IL-17-IFN-γ- cells among GM-CSF+CD4+ dLN T lymphocytes from AO rats, the expression of mRNA for IL-7, the cytokine suggested to drive Th-GM cell differentiation in mice and possibly rats [19,28] was diminished (p≤0.001) in dLN tissue from AO (0.63±0.004) compared with DA rats (1.00±0.042). This could be linked with the lower number of dLN cells and lower (p≤0.01) frequency of IL-17-IFN-γ-GM-CSF+CD4+ T lymphocytes among them in AO (7.89±0.23%) compared with DA rats (9.53±0.31%).

### Different frequency of inflammatory monocytes among peripheral blood cells from AO and DA rats on the 7^th^ d.p.i.

**Fig 8:**
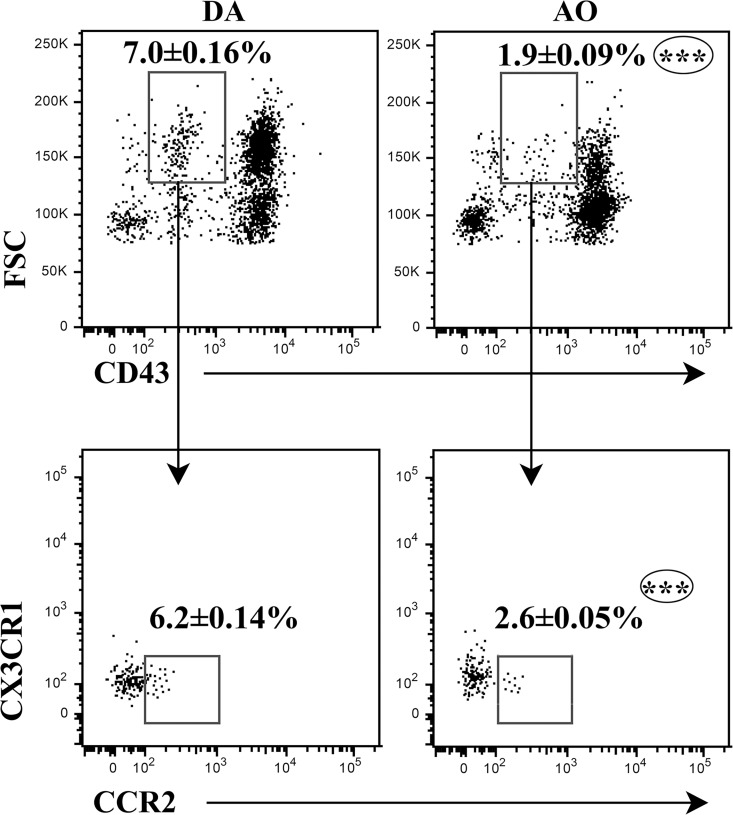
Lower frequency of CCR2+CX3CR1- cells within large CD43^low^ monocytes in peripheral blood of AO compared with DA rats immunized for EAE. Flow cytometry dot plots show the frequency of (lower) CCR2+CX3CR1- cells within (upper) large CD43^low^ peripheral blood monocytes retrieved from DA and AO rats on the 7^th^ d.p.i. Data (mean ± SEM) are representative of two experiments (n = 6). *** p≤0.001.

Given that GM-CSF accelerates the release of bone marrow myeloid cells, which infiltrate the CNS and ultimately differentiate into inflammatory macrophages and dendritic cells [23], the frequency of large inflammatory monocytes exhibiting CD43^low^CCR2+CX3CR1- phenotype [46,47] was explored. The frequencies of large CD43^low^ cells among peripheral blood cells, and CCR2+CX3CR1- cells among them were lower (p≤0.001) in AO than in DA rats (Fig 8).

Differently from classical CD43^high^ monocytes, rat inflammatory CD43^low^ monocytes also express CD11b at high levels (CD11b^hi^) and CD32, as well as CD62L and CCR7 [46,47]. In agreement with the previous finding, the frequency of CD11b^hi^ cells, as well those of CD32+ and CCR7+CD62L+ cells among them were also lower (p≤0.01) in peripheral blood from AO rats (S8 Fig).

### Different phenotypic profile of CD4+ T lymphocytes and CD11b+ non-lymphoid cells from AO and DA rat SC on the 7^th^ d.p.i.

#### CD4+ T lymphocytes

**Fig 9:**
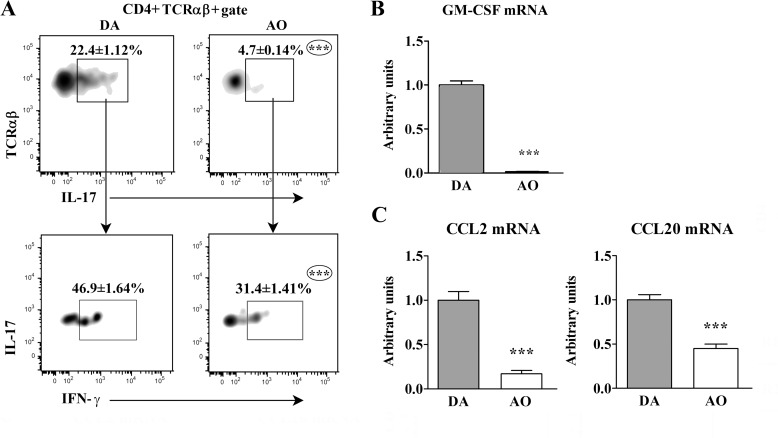
Lower frequency of all IL-17+ cells within CD4+TCRαβ+ lymphocytes and IFN-γ-producing cells within them in spinal cord of AO than DA rats immunized for EAE. **(A)** Flow cytometry density plots show the frequency of (upper) IL-17+ cells within CD4+TCRαβ+ lymphocytes and (lower) IFN-γ-co-producing (IL-17+IFN-γ+) cells within IL-17+CD4+TCRαβ+ lymphocytes retrieved from spinal cord (SC) of DA and AO rats on the 7^th^ d.p.i. **(B)** Fold change in GM-CSF mRNA expression in SC cells of AO rats relative to those cells from their DA counterparts. **(C)** Fold change in CCL2 and CCL20 mRNA expression in SC tissue retrieved from AO rats on the 7^th^ d.p.i. relative to SC tissue from their DA counterparts. Data (mean ± SEM) are representative of two experiments (n = 6). *** p≤0.001.

Although similar number of mononuclear cells was retrieved from SC of AO and DA rats, lower (p≤0.001) number of CD4+ T lymphocytes was found among them in AO compared with DA rats (S9 Fig). Besides, the frequency of IL-17+ cells in PMA- and ionomycine-stimulated CD4+ T lymphocytes from AO rat SC was lower (p≤0.001) than in those cells from DA rat SC (Fig 9A). Moreover, compared with DA, in AO rats the frequency of IL-17+IFN-γ+ cells was lower (p≤0.001) among IL-17+ CD4+ T lymphocytes (Fig 9A). Consistently, the amount of GM-CSF mRNA was markedly diminished (p≤0.001) in mononuclear SC cells from AO compared with DA rats (Fig 9B). The lower frequency of all IL-17+ in CD4+ T lymphocytes and of IL-17+IFN-γ+ cells among them was consistent with the lower (p≤0.001) expression of CCL2 and CCL20 mRNA in SC from AO compared with DA rats (Fig 9C).

#### CD11b+ non-lymphoid cells

**Fig 10:**
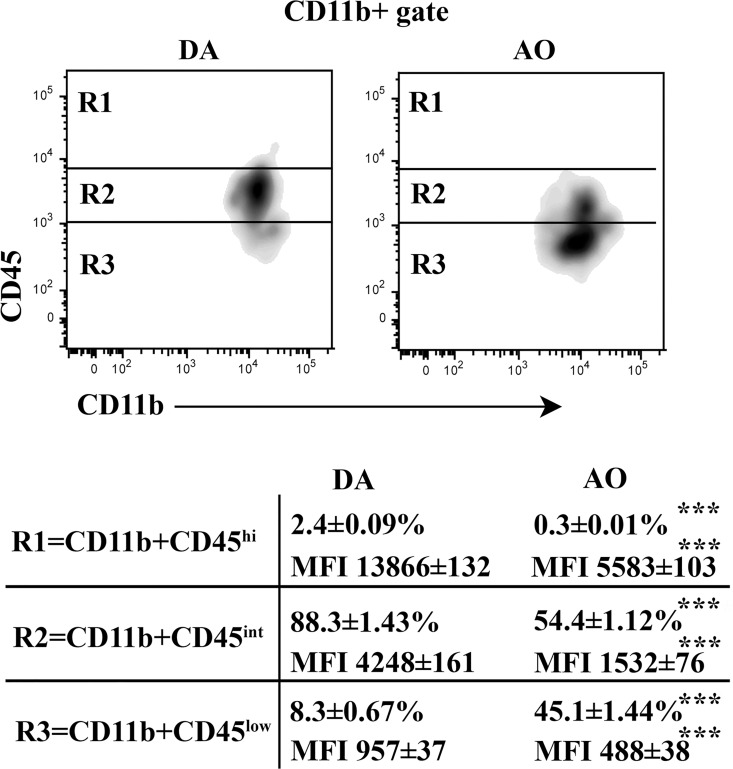
Lower frequency of CD45^hi^ and CD45^int^ cells within CD11b+ cells in spinal cord of AO compared with DA rats immunized for EAE. Flow cytometry density plots represent CD45 staining of CD11b+ spinal cord (SC) cells retrieved from DA and AO rats on the 7^th^ d.p.i. Table indicates the frequency of cells and CD45 mean fluorescence intensity (MFI) on CD45+ CD11b+ SC cells in the indicated region (R). Data (mean ± SEM) are representative of two experiments (n = 6). *** p≤0.001.

Given that the inflammatory monocyte migration into the CNS is regulated by production of CCL2 by CNS cells [48], the frequency of monocyte-derived cells was examined. Differential CD45 staining intensity coupled with CD11b staining has been used to distinguish between infiltrating inflammatory monocytes progeny, activated and non-activated microglia [49,50]. The monocyte-derived cells have been suggested to predominantly express CD45 at high levels (CD45^hi^ cells) [50]. Setting the boundaries between CD11b+ cells expressing intermediate (CD45^int^ cells; mainly activated microglial cells, although some monocyte derived cells also exhibit this phenotype) and high levels of CD45 as previously described [41], we found that the frequency of CD45^hi^ cells was considerably lower (p≤0.001) in the CD11b+ population retrieved from AO than DA rat SC (Fig 10). Additionally, the frequency of CD45^int^ cells was lower (p≤0.001) among CD11b+ cells from AO than DA rat SC (Fig 10).

### Different phenotypic profile of CD4+ T lymphocytes and CD11b+ non-lymphoid cells from AO and DA rat SC on the 13^th^ d.p.i.

#### CD4+ lymphocytes

**Fig 11:**
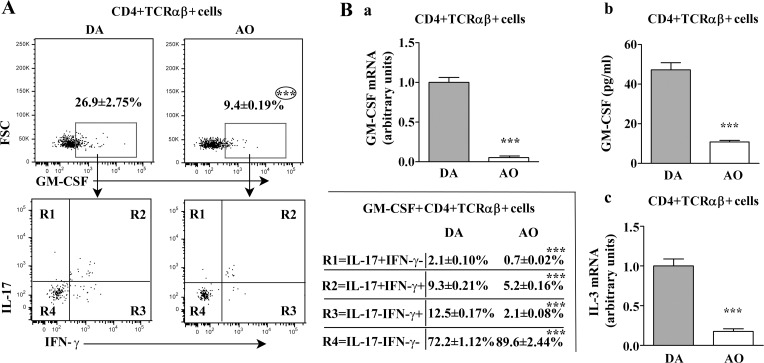
Lower frequency of GM-CSF+ cells within CD4+TCRαβ+ lymphocytes in spinal cord of AO compared with DA rats immunized for EAE. **(A)** Flow cytometry dot plots show the frequency of (upper) GM-CSF+ cells within CD4+TCRαβ+ lymphocytes and (lower) the frequency of their distinct subsets delineated according to IL-17/IFN-γ expression in spinal cord (SC) of DA and AO rats on the 13^th^ d.p.i. CD4+TCRαβ+ lymphocytes were separated using magnetic-activated cell sorting (MACS) as described in Materials and Methods. The gating strategy is displayed in S11 Fig. Table indicates frequency of cells in the indicated region (R). **(B)** Fold change in GM-CSF mRNA expression in CD4+TCRαβ+ SC cells retrieved from AO rats on the 13^th^ d.p.i. relative to those cells from DA rats (a). Concentration of GM-CSF in supernatants of PMA/ionomycine stimulated CD4+TCRαβ+ lymphocytes retrieved from SC of DA and AO rats on the 13^th^ d.p.i. (b). Fold change in IL-3 mRNA expression in CD4+TCRαβ+ lymphocytes retrieved from SC of AO rats on the 13^th^ d.p.i. relative to those cells from their DA counterparts (c). Data (mean ± SEM) are representative of two experiments (n = 6). *** p≤0.001.

To discern any differences in infiltrating CD4+ T lymphocyte composition on the 13^th^ d.p.i., FCA on SC mononuclear cells was performed. Compared with DA rats, dramatically fewer (p≤0.001) mononuclear cells were retrieved from AO rat SC, and they contained fewer (p≤0.001) CD4+ T lymphocytes (S10 Fig). The immunophenotyping results showed lower (p≤0.001) frequency of GM-CSF+ cells among PMA- and ionomycine-stimulated CD4+ T lymphocytes from AO rats in respect to DA rats (Fig 11A). Consistently, CD4+ T lymphocytes from AO rats contained less (p≤0.001) amount of GM-CSF mRNA than those from DA rats (Fig 11Ba). Besides, the concentration of GM-CSF was lower (p≤0.001) in PMA-and ionomycine-stimulated CD4+ T lymphocyte cultures from AO than DA rat SC (Fig 11Bb). The immunophenotyping of GM-CSF+ CD4+ T lymphocytes infiltrating SC showed higher (p≤0.001) frequency of IL-17-IFN-γ- cells, but lower (p≤0.001) frequencies of IL-17-IFN-γ+, IL-17+IFN-γ- and IL-17+IFN-γ+ cells in AO compared with DA rats (Fig 11A). However, as in dLNs, given that the frequency of IL-17-IFN-γ-GM-CSF+ cells among CD4+ T lymphocytes was lower (p≤0.001) in AO (8.64±0.20%) than in DA rat SC infiltrate (19.98±0.52%), less (p≤0.001) amount of IL-3 mRNA was detected in CD4+ T lymphocytes from AO rats (Fig 11Bc).

**Fig 12:**
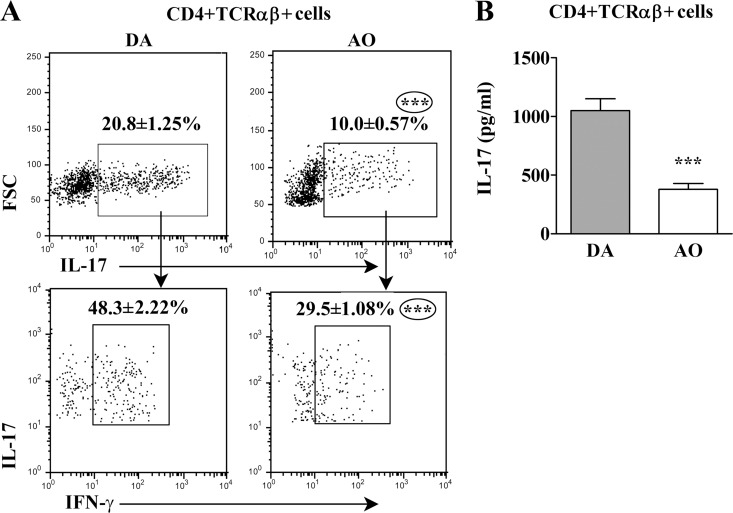
Lower frequency of IL-17+ cells within CD4+TCRαβ+ lymphocytes in spinal cord of AO compared with DA rats immunized for EAE. **(A)** Flow cytometry dot plots show the frequency of (upper) IL-17+ cells within CD4+TCRαβ+ lymphocytes and (lower) the frequency of IL-17+IFN-γ+ cells within them in spinal cord (SC) of DA and AO rats on the 13^th^ d.p.i. **(B)** Concentration of IL-17 in supernatants of PMA/ionomycine-stimulated CD4+TCRαβ+ lymphocytes retrieved from SC of DA and AO rats on the 13^th^ d.p.i. Data (mean ± SEM) are representative of two experiments (n = 6). *** p≤0.001.

Among PMA- and ionomycine-stimulated CD4+ T lymphocytes from AO rat SC, lower (p≤0.001) frequency of IL-17+ cells was also found when compared with DA rats (Fig 12A). Besides, the frequency of IL-17+IFN-γ+ cells was also lower (p≤0.001) among IL-17+ CD4+ T lymphocytes from AO than DA rat SC (Fig 12A). Furthermore, the concentration of IL-17 was lower (p≤0.001) in PMA- and ionomycine-stimulated mononuclear SC cells from AO than DA rats (Fig 12B).

**Fig 13:**
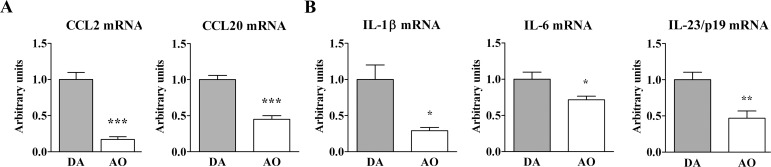
Lower expression of chemokines and cytokines in spinal cord of AO than DA rats immunized for EAE. **(A)** Fold change in CCL2 and CCL20 mRNA expression in spinal cord tissue of AO rats on the 13^th^ d.p.i. relative to spinal cord (SC) tissue of their DA counterparts. **(B)** Fold change in IL-1β, IL-6 and IL-23/p19 mRNA expression in SC cells retrieved from AO rats on the 13^th^ d.p.i. relative to those cells from their DA counterparts. Data (mean ± SEM) are representative of two experiments (n = 6). * p≤0.05; ** p≤0.01; *** p≤0.001.

Consistently, CCL2 and CCL20 mRNA expression were lower (p≤0.001) in AO than DA rat SC (Fig 13A). Next, SC expression of mRNAs for the key cytokines coordinating differentiation of Th17 cells from naive precursors and their acquisition of IL-17+IFN-γ+ phenotype was examined. The expression of mRNAs for IL-1β (p≤0.05), IL-6 (p≤0.05) and IL-23/p19 (p≤0.01) was lower in mononuclear SC cells from AO compared with DA rats (Fig 13B). TGF-β mRNA expression did not differ between these cells from AO (0.96±0.143) and DA rats (1.00±0.011).

The expression of mRNA for IL-7, the cytokine suggested to drive generation of Th-GM cells in rodents [19,28] was also examined. The lower (p≤0.001) IL-7 mRNA expression in AO (0.47±0.019) than DA rat (1.00±0.073) was consistent with the lower number of CD4+ T lymphocytes and the frequency of IL-17-IFN-γ-GM-CSF+ cells among them in AO rat compared with DA rat SC.

#### CD11b+ non-lymphoid cells

**Fig 14:**
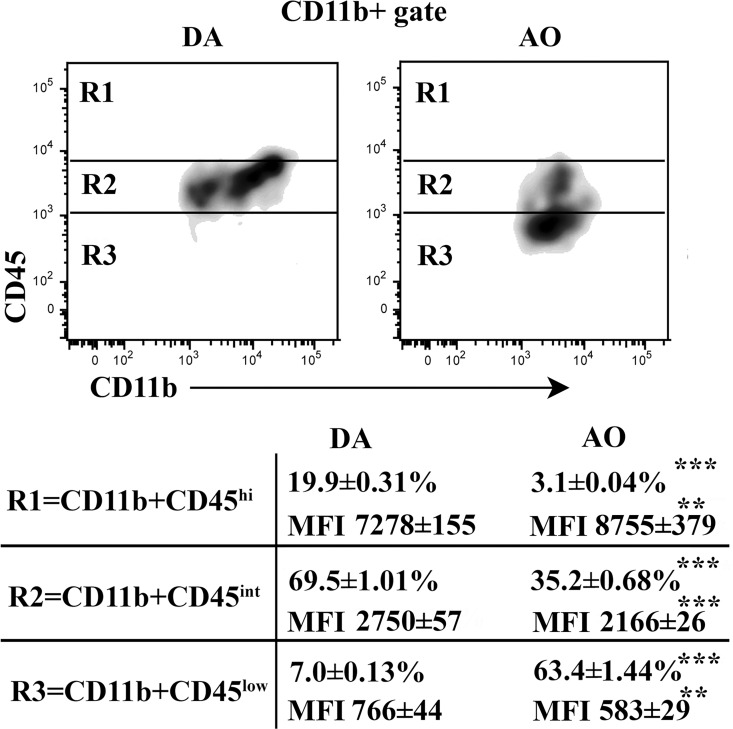
Lower frequency of CD45^hi^ and CD45^int^ cells within spinal cord CD11b+ cells from AO than DA rats immunized for EAE. Flow cytometry density plots indicate CD45 staining of CD11b+ spinal cord (SC) cells retrieved from DA and AO rats on the 13^th^ d.p.i. Table indicates the frequency of cells and CD45 mean fluorescence intensity (MFI) on CD45+ CD11b+ SC cells in the indicated region (R). Data (mean ± SEM) are representative of two experiments (n = 6). ** p≤0.01; *** p≤0.001.

Furthermore, on the 13^th^ d.p.i., CD11b+ cells from SC were immunophenotyped. Compared with DA rats, in AO rats was found lower (p≤0.001) frequency of CD45^hi^ cells, which are supposed to represent mainly monocyte-derived cells [50] (Fig 14). Besides, among CD11b+ cells the frequency of CD45^int^ cells, corresponding mainly to activated microglial cells [50], was also lower (p≤0.001) in AO compared with DA rats (Fig 14).

## Discussion

The study showed diminished and qualitatively altered response of CD4+ dLN T lymphocytes to the inoculation of emulsion of SC tissue homogenate and CFA in AO rats compared with DA ones. Specifically, fewer activated CD134+CD4+ T lymphocytes were retrieved from AO than DA rat dLNs. Given that, judging by CD40 MFI [51], antigen presenting CD40+CD11b+CD45RA- cells from AO rats exhibited stronger activation than those from DA rats, and that CD4+ T:CD40+CD11b+CD45RA- cell ratio was shifted towards the antigen presenting cells in AO rats (56.1±1.21 in AO rats vs 81.25±5.46 in DA rats), whereas the frequency of CD25+FoxP3+ cells among CD4+ T lymphocytes was comparable between these two rat strains, the strain-specific differences in CD4+ lymphocyte proliferative capacity may be assumed. In favor of this assumption are data from previous studies indicating diminished production of IL-2 by dLN lymphocytes from AO compared with DA rats [52].

In addition to lower generation of neuroantigen-specific CD4+ T lymphocytes in AO rat dLNs, the present study suggests impaired polarization of CD4+ T lymphocytes towards GM-CSF+ Th cells, as their frequency was reduced among both PMA/ionomycine- and MBP-stimulated CD4+ T lymphocytes from AO compared with DA rats. Consistently, the concentration of GM-CSF was lower in MBP-stimulated dLN cell cultures from AO rats. GM-CSF is necessary to render CD4+ T lymphocytes pathogenic in mouse models of EAE [17,18], so GM-CSF^−/−^ mice are resistant to EAE [53]. Thus, it may be assumed that the diminished frequency of GM-CSF+ cells among neuroantigen-specific CD4+ T lymphocytes additionally contributed to AO rat resistance to EAE induction. To corroborate this notion is our previous study indicating that in AO rats the age-related increase in susceptibility to EAE coincides with the augmented generation of neuroantigen-specific GM-CSF+ Th cells in dLNs and their greater frequency in the SC [28]. In the same line is higher concentration of GM-CSF in MBP-stimulated dLN cell cultures and in cultures of PMA/ionomycine-stimulated CD4+ T cells from SC of aged (24-26-month-old) AO rats immunized for EAE compared with the corresponding cultures from their younger (2-3-month-old) counterparts (S12 Fig). On the contrary, the lower concentration of this cytokine was found in PMA/ionomycine-stimulated SC mononuclear cell cultures from aged DA rats immunized for EAE (21.31±0.21 pg/ml) than in those from their young counterparts (48.01±0.31 pg/ml) exhibiting markedly higher EAE incidence and greater neurological deficit, further supporting a putative role of GM-CSF in rat susceptibility to clinical EAE.

The immunophenotyping of GM-CSF+CD4+ T lymphocytes confirmed their phenotypic heterogeneity [28]. It has recently been shown that the autoimmune inflammation in mice can be driven by phenotypically distinct GM-CSF+ Th cell subsets belonging to separate cell lineages [54–56]. Specifically, Th-GM cells, belonging to separate lineage [19] are suggested to cooperate with IL-17+IFN-γ+ Th17 and/or Th1 cells to induce autoimmune (neuro)inflammation in mice [55–57]. Our results suggest that compared with DA rats, in AO rats Th-GM lymphocyte differentiation in dLNs was favored on the account of differentiation of multi-cytokine-producing highly pathogenic GM-CSF+ IFN-γ+ Th17 cells. The diminished expression of mRNAs for not only IL-6 and IL-23/p19, as previously shown [58], but also IL-1β, i.e. the cytokines driving their differentiation [43–45] in dLN cells from AO rats additionally corroborates the previous notion. These findings are also in agreement with those obtained in our previous study indicating differential polarization capacity of splenic dendritic cells from AO and DA rats [59]. The lower frequency of IFN-γ+ cells among GM-CSF+ CD4+ T lymphocytes could also support the diminished differentiation of their IL-17+IFN-γ+ predecessors, as they are shown to represent Th17 cells that lost IL-17 expression, i.e. ex-Th17 cells [55,56]. However, it should be pointed that they may also belong to a separate cell lineage [54].

In agreement with the lower production of GM-CSF by CD4+ dLN T lymphocytes, lower frequency of inflammatory monocytes [25,26] was found in peripheral blood from AO compared with DA rats. Given that their enrichment in the circulating pool is associated with an earlier onset and increased severity of clinical EAE [25], this finding is also consistent with the resistance of AO rats to EAE.

Given that the factors influencing pathogenic Th cell homing into the CNS also influence EAE development [37], Th17 cells and their IL-17+IFN-γ+ subset were examined for the expression of CCR6 and CCR2. The expression of these chemokine receptors is temporally regulated during CD4+ lymphocyte priming, so that the appearance of earliest classical Th17 cells, predominantly expressing CCR6, is followed by later emergence of pathogenic GM-CSF+IFN-γ+ CCR6-CCR2+ Th cells [37]. In rats, as in mice [37,42], almost all IL-17+IFN-γ+ Th17 cells exhibited surface expression of CCR2, but its density was slightly lower on the multi-cytokine producing Th17 cells from AO rats. Differently, in agreement with data obtained in mice [37], CCR6 was observed on great majority of IL-17+ cells and the frequency of CCR6-expressing cells among IL-17+ CD4+ T cells and CCR6 surface density on these cells were comparable in AO and DA rats. These findings, coupled with the markedly lower expression of CCL2 and CCL20 in SC from AO compared with DA rats suggest impaired homing of conventional and highly pathogenic multi-cytokine-producing Th17 cells into the SC of AO rats. Besides, the lower frequency of all IL-17+ cells among CD4+ T lymphocytes, and IL-17+IFN-γ+ cells among them, in SC of AO compared with DA rats, could be associated with the reduced expression of mRNAs for the major cytokines driving their differentiation, i.e. IL-1β, IL-6 and IL-23/p19. Comparable expression of TGF-β mRNA in mononuclear SC cells from AO and DA rats is consistent with data indicating that in mouse EAE models, independently of TGF-β, IL-6, IL-1β and IL-23 not only efficiently generate Th17 cells from their naïve precursors, but also promote generation of the pathogenic cells co-expressing IL-17 and IFN-γ [45]. The frequency of the latter cells was also reduced among GM-CSF+ CD4+ T lymphocytes from AO rats. Given that elegant fate-mapping studies showed that IL-17-IFN-γ+ GM-CSF+ cells correspond to the cells which ultimately extinguish IL-17 expression [55,56], their lower frequency among GM-CSF+ CD4+ T lymphocytes from AO compared with DA rat SC is consistent with the lower frequency of IL-17+IFN-γ+ cells (supposed to be their predecessors) within this subpopulation from AO rats. However, the contribution of GM-CSF+IFN-γ+ cells belonging to separate cell lineage [54] to the lower frequency of IL-17-IFN-γ+ cells among GM-CSF+ CD4+ T lymphocytes could also not be ruled out. Moreover, higher frequency of IL-17-IFN-γ- cells among GM-CSF+CD4+T lymphocytes infiltrating AO rat SC, additionally supports the differential regulation of CD4+ T lymphocyte differentiation in AO and DA rats SC.

Consistent with the diminished frequency of inflammatory monocytes among peripheral blood cells in AO compared with DA rats, and the lower production of GM-CSF by CD4+ T lymphocytes infiltrating AO rat SC (reflecting the lower frequency of GM-CSF+ cells among them), diminished frequency of CD11b+CD45^hi^ cells, corresponding mainly to monocyte-derived inflammatory macrophages and dendritic cells [50] was found in mononuclear SC cell suspensions from AO rats. Several data indicate that this correlation is not coincidental, but causal. Firstly, in the mouse, circulating CCR2+Ly6C^hi^ monocytes, which correspond to rat CD43^low^CCR2+CX3CR1- cells [46,47], traffic across the blood-brain barrier, up-regulate proinflammatory molecules, and differentiate into macrophages and dendritic cells with high damaging capacity [25,26]. Secondly, GM-CSF not only accelerates the release of bone marrow precursors, which in EAE ultimately differentiate into CNS-infiltrating inflammatory macrophages and dendritic cells [23], but also controls the expression of a pathogenic signature in CCR2+Ly6C^hi^ monocytes and their progeny, which is essential for tissue damage [25,26]. However, to the impaired inflammatory monocyte infiltration into the SC of AO compared with DA rats, apart from their diminished release from bone marrow due to the lower GM-CSF production, the reduced CCL2 mRNA expression in AO rat SC could also contribute [23]. Using a combination of parabiosis and myeloablation to replace circulating progenitors without affecting CNS-resident microglia, a strong correlation between monocyte infiltration and progression to the paralytic stage of EAE was found [60]. Additionally, it has been shown that CCR2^–/–^mice do not develop clinical EAE or CNS histopathology [61].

Moreover, given that GM-CSF is shown to be the major stimulator of microglia proliferation and activation [62,63], the lower frequency of CD11b+CD45^int^ cells, mainly belonging to activated microglia [50] on the 13^th^ d.p.i. was consistent with the lower production of GM-CSF from Th cells infiltrating AO than DA rat SC.

In conclusion, the study advances our understanding of immunological differences between AO and DA rats [39,58] indicating that the resistance of AO rats to EAE could be related to: i) lower generation of highly pathogenic neuroantigen-specific GM-CSF+ Th cells in their dLNs (most likely due to intrinsic defect in their proliferative capacity and the diminished microenvironment ability to support differentiation of highly pathogenic multi-cytokine-producing GM-CSF+IFN-γ+ Th17 cells) when compared with DA rats sensitive to EAE induction, and ii) impaired SC expression of chemokines driving migration of neuroantigen-specific conventional and the multi-cytokine producing GM-CSF+IFN-γ+ Th17 cells (i.e. CCL20 and CCL2 respectively) into the SC, whose microenvironment is also less supportive of differentiation of pathogenic multi-cytokine-producing Th17 cells. Thus, the study further promotes: i) role of Th-cell-derived GM-CSF and SC cell-derived chemokines in development of autoimmune neuroinflammation, and ii) these molecules as putative targets in therapy of MS, and most likely some other autoimmune inflammatory diseases. In addition, it indicates that multiple rather than single genetically-determined immunological difference underlie strain differences in susceptibility to clinical EAE and possibly individual variations in clinical presentation of MS, and thereby provides scientific basis for understanding almost axiomatic individual variations in responses to MS therapies, particularly those targeting single molecule/mechanism [64].

## Supporting Information

S1 Fig Strain differences in neurological signs of EAE. **(A)** Line graph illustrates the monophasic EAE course in DA rats and the absence of neurological signs of the disease in AO rats immunized with spinal cord homogenate in in phosphate-buffered saline supplemented with complete Freund’s adjuvant and injected with for EAE. Rats were examined for neurological signs of the disease daily, from the 7^th^ day post-immunization (d.p.i.) until the 21^st^ d.p.i.. Data (mean ± SEM) were obtained from preliminary experiment which included 10 rats per group. Note that none of rats reached score 4 (tetraplegia or moribund state). **(B)** Line graph indicates daily clinical score of EAE in DA and AO rats from the 7^th^ to the 13^th^ d.p.i. **(C)** Scatter plot indicates maximal clinical sign of EAE until the 13^th^ d.p.i. The incidence of EAE in DA rats was 100% whereas none of AO rats exhibited neurological signs of the disease. Data (mean ± SEM) are representative of two experiments (n = 12).
(TIF)

S2 Fig Lower expression of MHC II on CD11b+CD45RA- cells retrieved from draining lymph nodes of AO than DA rats immunized for EAE. Lower flow cytometry dot plots show the frequency of MHC II+ cells within CD11b+CD45RA- cells gated on draining lymph node (dLN) cells retrieved from of DA and AO rats on the 7^th^ day post-immunization (d.p.i.) as shown in the upper flow cytometry dot plots. This gating strategy was used for CD11b+CD45RA- cells in Fig 1. Numbers in the flow cytometry dot plots indicate the frequency of (upper) CD11b+CD45RA- cells and (lower) MHC II+ cells within them and MHCII mean fluorescence density (MFI) on MHC II+ cells. Bar graph represents the number of CD11b+CD45RA-MHC II+ cells retrieved from dLNs of DA and AO rats on the 7^th^ d.p.i. Data (mean ± SEM) are representative of two experiments (n = 6). ** p≤0.001; *** p≤0.001.
(TIF)

S3 Fig Gating strategy for flow cytometry analysis of *in vitro* proliferating CD4+ lymphocytes from draining lymph nodes of DA and AO rats immunized for EAE. **(A)** Flow cytometry dot plots indicate gating strategy for cultivated CD4+ draining lymph node (dLN) lymphocytes retrieved from DA and AO rats on the 7^th^ day post-immunization (d.p.i.) **(B)** Flow cytometry histograms indicate 7-AAD staining of CD4+ lymphocytes retrieved from DA and AO rat dLNs on the 7^th^ d.p.i. and cultured (upper) in RPMI alone or in RPMI supplemented with (middle) ConA or (lower) MBP. The frequency of proliferating cells (cells in S+G2/M phases of cell cycle) was determined using the Dean-Jet-Fox model of the cell cycle platform generated by FlowJo software and displayed in Fig 2.
(TIF)

S4 Fig Comparable frequencies of CD25+FoxP3+ cells within CD4+ cells in draining lymph nodes of DA and AO rats immunized for EAE. Flow cytometry dot plots represent CD25 vs FoxP3 staining of CD4+ draining lymph node lymphocytes retrieved from DA and AO rats on the 7^th^ day post-immunization. Numbers in the flow cytometry dot plots indicate the frequency of CD25+FoxP3+ cells within CD4+ lymphocytes. Data (mean ± SEM) are representative of two experiments (n = 6).
(TIF)

S5 Fig IL-4 production inTCRαβ+ lymphocytes from draining lymph node of DA and AO rats immunized for EAE. Flow cytometry dot plots represent IL-4 vs TCRαβ staining of draining lymph node cells retrieved from DA and AO rats on the 7^th^ day post immunization and *in vitro* stimulated with PMA and ionomycine (as described in Materials and Methods). Note the absence of IL-4 staining in TCRαβ+ lymphocytes from rats of both strains. Data (mean ± SEM) are representative of two experiments (n = 6).
(TIF)

S6 Fig Fluorescence minus one controls for flow cytometry analyses of GM-CSF/IL-17/IFN-γ staining of CD4+TCRαβ+ lymphocytes retrieved from draining lymph nodes of rats immunized for EAE. The gating strategy for distinct subsets (delineated according to IL-17/IFN-γ expression) of GM-CSF+ CD4+TCRαβ+ lymphocytes retrieved from draining lymph nodes of rats on the 7^th^ day post-immunization (shown in **D)** is based upon fluorescence minus one controls: **(A)** minus GM-CSF, **(B)** minus IL-17 and **(C)** minus IFN-γ. CD4+TCRαβ+ lymphocytes were separated using magnetic-activated cell sorting (MACS) as described in Materials and Methods. This gating strategy was used in Fig 3.
(TIF)

S7 Fig Gating strategy and fluorescence minus one controls for flow cytometry analysis of CCR2/IL-17/IFN-γ staining of CD4+TCRαβ+ lymphocytes from draining lymph nodes retrieved from DA and AO rats immunized for EAE. The gating strategy for CCR2-expressing IL-17+IFN-γ+ CD4+TCRαβ+ lymphocytes retrieved from draining lymph nodes of rats on the 7^th^ day post-immunization (shown in **D)** is based upon fluorescence minus one controls: **(A)** minus IL-17, **(B)** minus IFN-γ and **(C)** minus CCR2. CD4+TCRαβ+ lymphocytes were separated using magnetic-activated cell sorting (MACS) as described in Materials and Methods. This gating strategy was used in Fig 5.
(TIF)

S8 Fig Lower frequency of CD32+ cells and CCR7+CD62L+ cells within large CD11b^hi^ monocytes in peripheral blood of AO than DA rats immunized for EAE. **(A)** Lower flow cytometry dot plots represent CD11b vs CD32 staining of CD11b^hi^ peripheral blood (PB) cells retrieved from DA and AO rats on the 7^th^ day post-immunization. The large CD11b^hi^ PB cells are gated as shown in the upper flow cytometry dot plots. Numbers in the flow cytometry dot plots indicate the frequency of (upper) large CD11b^hi^ cells within PB cells and (lower) CD32+ cells within CD11b^hi^ cells. **(B)** Flow cytometry dot plots indicate CD62L vs CCR7 staining of CD11b^hi^ PB cells of DA and AO rats. Numbers in the flow cytometry dot plots indicate the frequency of CCR7+CD62L+ cells within large CD11b^hi^ PB cells gated as indicated (A). Data (mean ± SEM) are representative of two experiments (n = 6). ** p≤0.01; *** p≤0.001.
(TIF)

S9 Fig Lower number of CD4+TCRαβ+ lymphocytes retrieved from spinal cord of AO than DA rats immunized for EAE on the 7^th^ day post-immunization. Bar graphs represent the number of (a) mononuclear cells (MNC) and (b) CD4+TCRαβ+ lymphocytes retrieved from spinal cord of DA and AO rats on the 7^th^ day post-immunization (d.p.i.). Data (mean ± SEM) are representative of two experiments (n = 6). *** p≤0.001.
(TIF)

S10 Fig Lower number of total mononuclear cells and CD4+TCRαβ+ lymphocytes retrieved from spinal cord of AO than DA rats immunized for EAE on the 13^th^ day post-immunization. Bar graphs represent the number of (a) mononuclear cells (MNC) and (b) CD4+TCRαβ+ lymphocytes from spinal cord of DA and AO rat immunized for EAE on the 13^th^ day post-immunization (d.p.i.). Data (mean ± SEM) are representative of two experiments (n = 6). *** p≤0.001.
(TIF)

S11 Fig Fluorescence minus one controls for flow cytometry analyses of GM-CSF/IL-17/IFN-γ staining of CD4+TCRαβ+ lymphocytes retrieved from spinal cord of rats immunized for EAE. The gating strategy for distinct subsets (delineated according to IL-17/IFN-γ expression) of GM-CSF+ CD4+TCRαβ+ lymphocytes retrived from spinal cord of rats on the 13^th^ day post-immunization (shown in **D)** is based upon fluorescence minus one controls: **(A)** minus GM-CSF, **(B)** minus IL-17 and **(C)** minus IFN-γ. CD4+TCRαβ+ lymphocytes were separated using magnetic-activated cell sorting (MACS) as described in Materials and Methods. This gating strategy was used in Fig 11.
(TIF)

S12 Fig Aging increases number of GM-CSF+CD4+TCRαβ+ lymphocytes retrieved from draining lymph nodes and spinal cord of AO rats immunized for EAE. **(A)** Bar graphs represent (a) the number of GM-CSF+CD4+TCRαβ+ cells retrieved from draining lymph nodes (dLNs) of young and aged AO rats on the 7^th^ day post-immunization (d.p.i.) and (b) concentration of GM-CSF in supernatants of dLN cells from young and aged AO rats cultured in RPMI alone or in the presence of MBP. **(B)** Bar graphs represent (a) the number of GM-CSF+CD4+TCRαβ+ cells retrieved from spinal cord (SC) of young and aged AO rats in the effector phase of the disease and (b) concentration of GM-CSF in supernatants of PMA/ionomycine stimulated CD4+TCRαβ+ lymphocytes retrieved from SC of young and aged AO rats. Data (mean ± SEM) are representative of two experiments (n = 9). * p≤0.05; *** p≤0.001; ### p≤0.001. * vs young AO rats; # vs RPMI.
(TIF)

S1 Table Summary of mRNA targets and reference gene for RT-qPCR analysis. (DOC)
