# Supplementary figures and images for: GM-CSF-Producing Th Cells in Rats Sensitive and Resistant to Experimental Autoimmune Encephalomyelitis
Source: PLoS One. 2016 Nov 10;11(11):e0166498. doi: 10.1371/journal.pone.0166498 (PMC5104330; doi:10.1371/journal.pone.0166498)

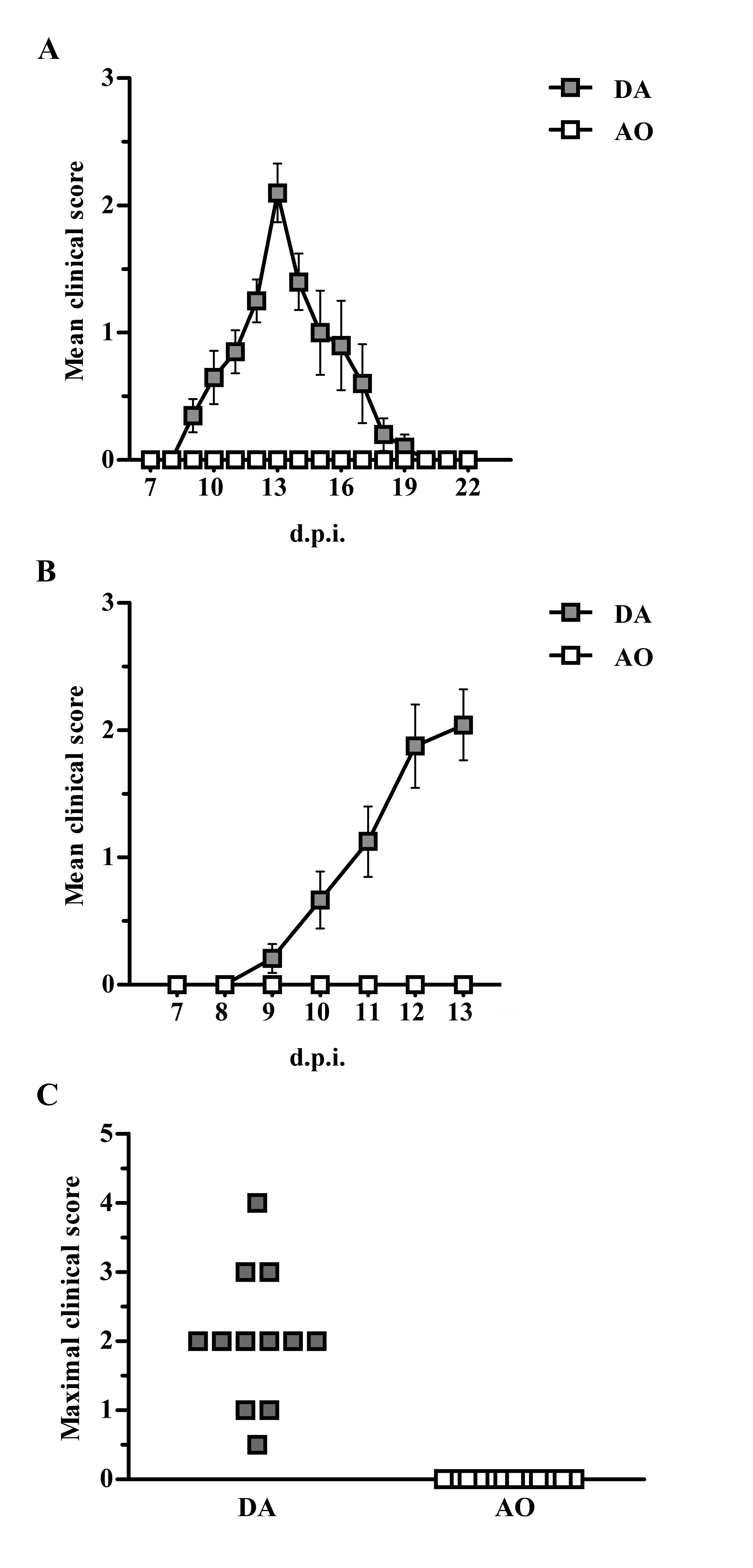

Supplement: S1 Fig — (A) Line graph illustrates the monophasic EAE course in DA rats and the absence of neurological signs of the disease in AO rats immunized with spinal cord homogenate in in phosphate-buffered saline supplemented with complete Freund’s adjuvant and injected with for EAE. Rats were examined for neurological signs of the disease daily, from the 7th day post-immunization (d.p.i.) until the 21st d.p.i.. Data (mean ± SEM) were obtained from preliminary experiment which included 10 rats per group. Note that none of rats reached score 4 (tetraplegia or moribund state). (B) Line graph indicates daily clinical score of EAE in DA and AO rats from the 7th to the 13th d.p.i. (C) Scatter plot indicates maximal clinical sign of EAE until the 13th d.p.i. The incidence of EAE in DA rats was 100% whereas none of AO rats exhibited neurological signs of the disease. Data (mean ± SEM) are representative of two experiments (n = 12). (TIF) [file pone.0166498.s001.tif]

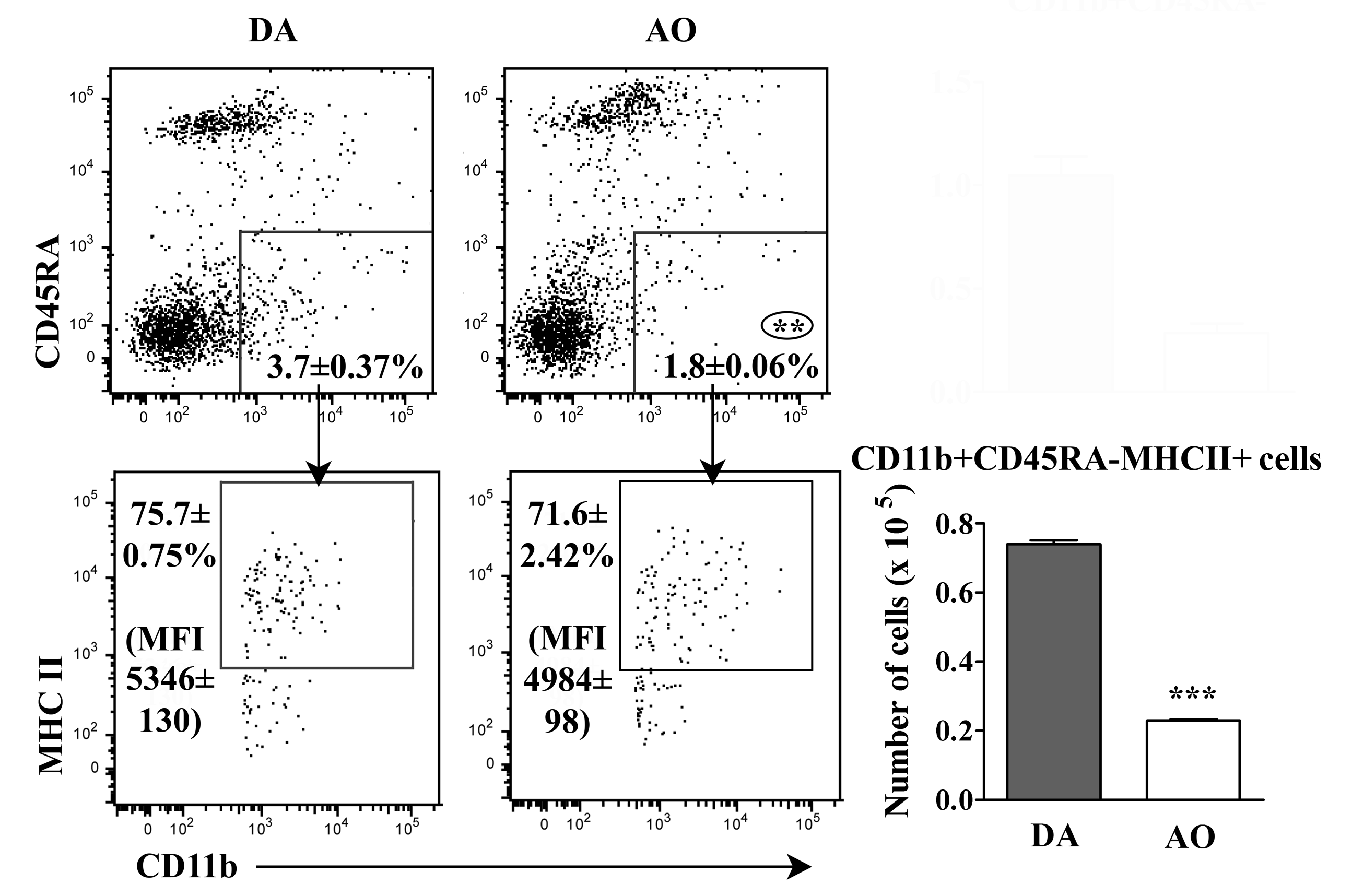

Supplement: S2 Fig — Lower flow cytometry dot plots show the frequency of MHC II+ cells within CD11b+CD45RA- cells gated on draining lymph node (dLN) cells retrieved from of DA and AO rats on the 7th day post-immunization (d.p.i.) as shown in the upper flow cytometry dot plots. This gating strategy was used for CD11b+CD45RA- cells in Fig 1. Numbers in the flow cytometry dot plots indicate the frequency of (upper) CD11b+CD45RA- cells and (lower) MHC II+ cells within them and MHCII mean fluorescence density (MFI) on MHC II+ cells. Bar graph represents the number of CD11b+CD45RA-MHC II+ cells retrieved from dLNs of DA and AO rats on the 7th d.p.i. Data (mean ± SEM) are representative of two experiments (n = 6). ** p≤0.001; *** p≤0.001. (TIF) [file pone.0166498.s002.tif]

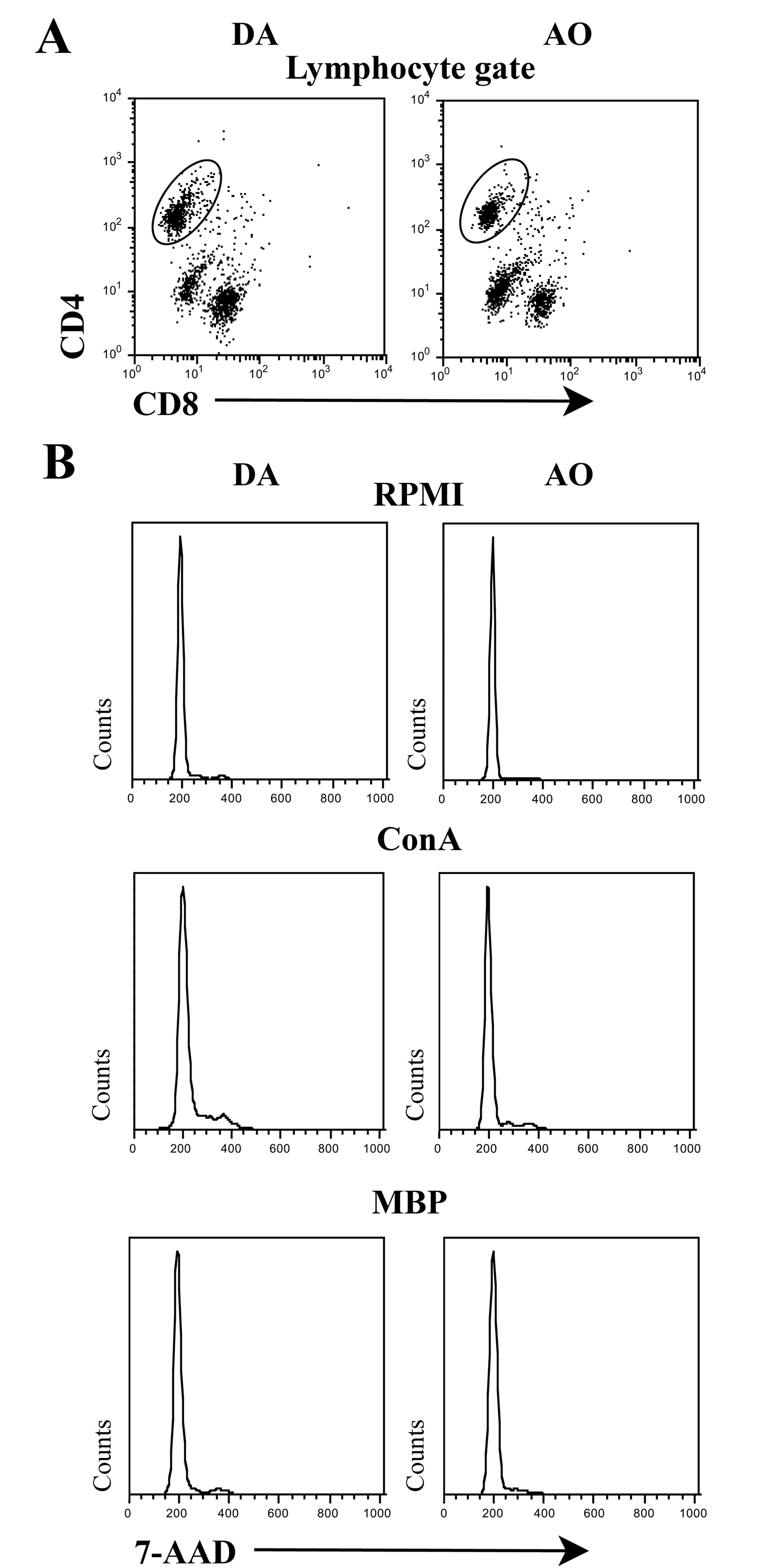

Supplement: S3 Fig — (A) Flow cytometry dot plots indicate gating strategy for cultivated CD4+ draining lymph node (dLN) lymphocytes retrieved from DA and AO rats on the 7th day post-immunization (d.p.i.) (B) Flow cytometry histograms indicate 7-AAD staining of CD4+ lymphocytes retrieved from DA and AO rat dLNs on the 7th d.p.i. and cultured (upper) in RPMI alone or in RPMI supplemented with (middle) ConA or (lower) MBP. The frequency of proliferating cells (cells in S+G2/M phases of cell cycle) was determined using the Dean-Jet-Fox model of the cell cycle platform generated by FlowJo software and displayed in Fig 2. (TIF) [file pone.0166498.s003.tif]

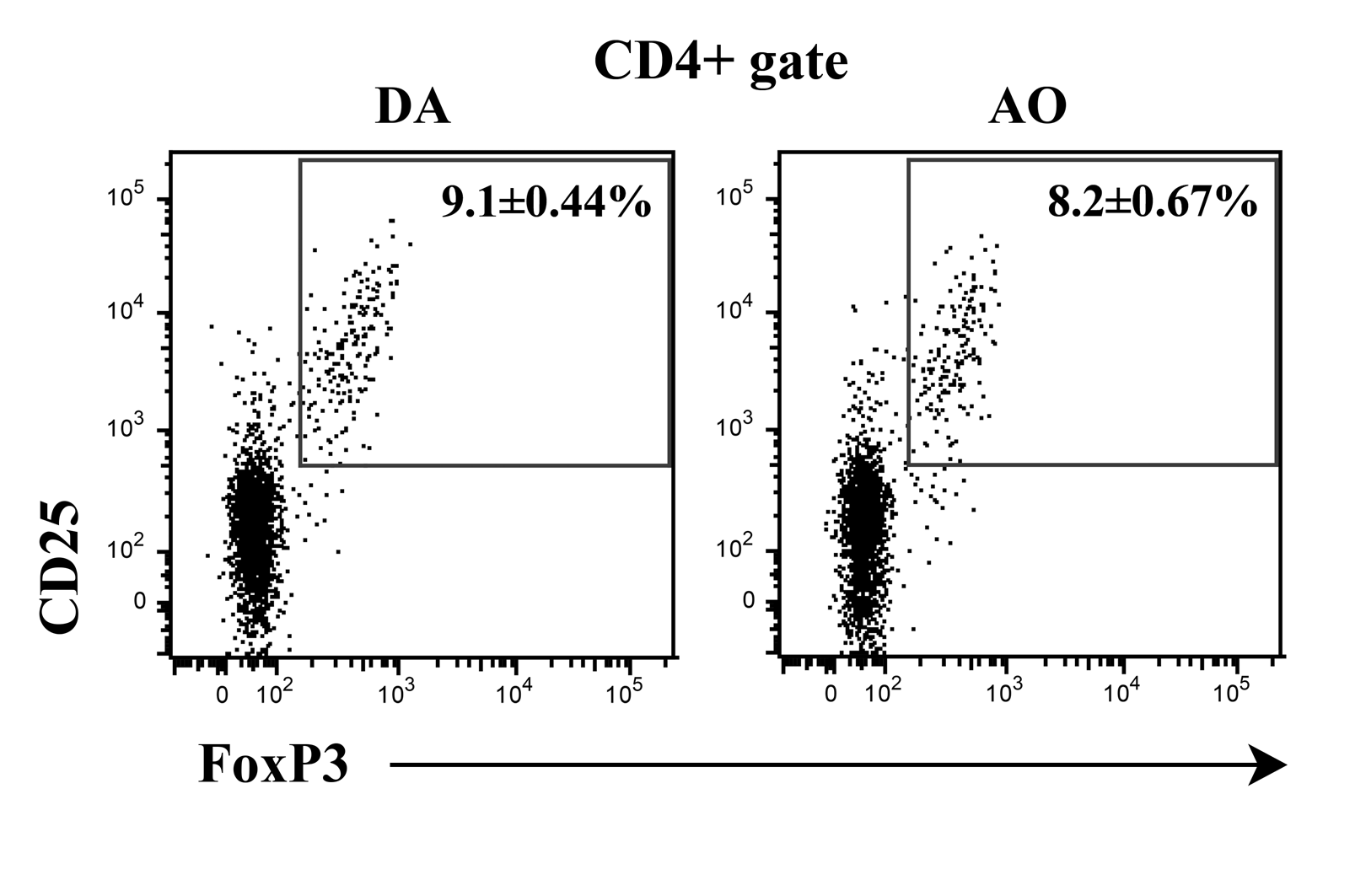

Supplement: S4 Fig — Flow cytometry dot plots represent CD25 vs FoxP3 staining of CD4+ draining lymph node lymphocytes retrieved from DA and AO rats on the 7th day post-immunization. Numbers in the flow cytometry dot plots indicate the frequency of CD25+FoxP3+ cells within CD4+ lymphocytes. Data (mean ± SEM) are representative of two experiments (n = 6). (TIF) [file pone.0166498.s004.tif]

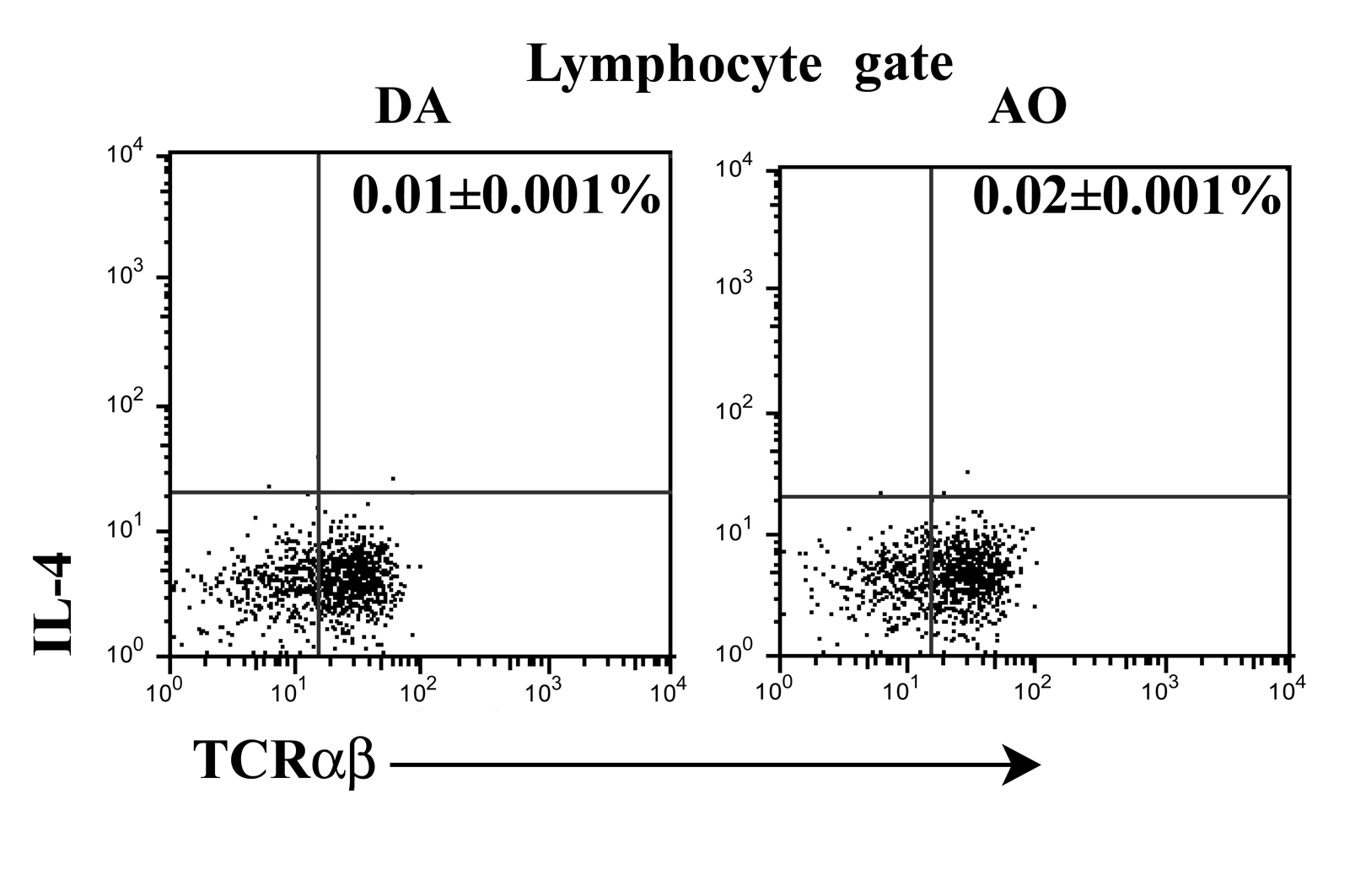

Supplement: S5 Fig — Flow cytometry dot plots represent IL-4 vs TCRαβ staining of draining lymph node cells retrieved from DA and AO rats on the 7th day post immunization and in vitro stimulated with PMA and ionomycine (as described in Materials and Methods). Note the absence of IL-4 staining in TCRαβ+ lymphocytes from rats of both strains. Data (mean ± SEM) are representative of two experiments (n = 6). (TIF) [file pone.0166498.s005.tif]

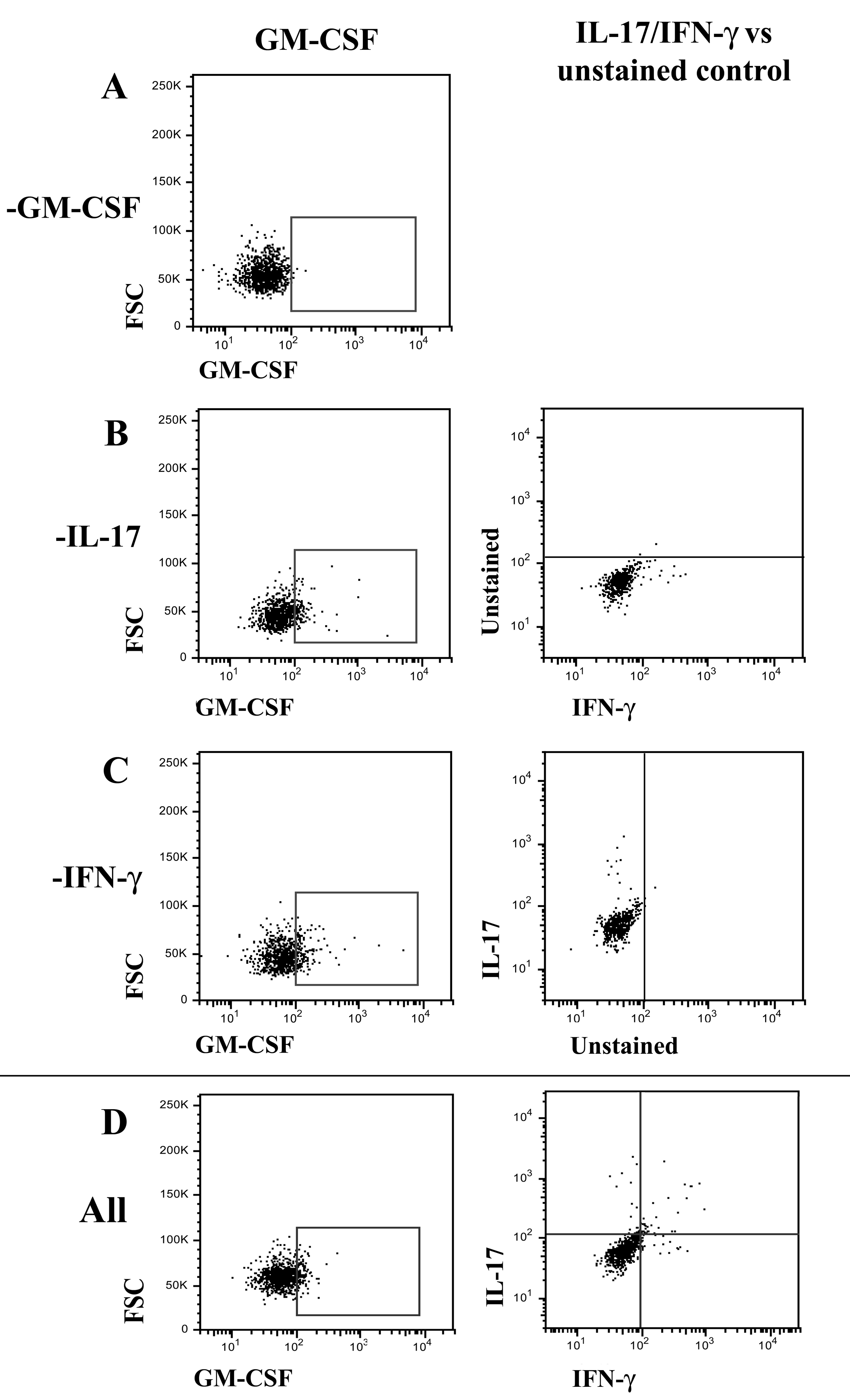

Supplement: S6 Fig — The gating strategy for distinct subsets (delineated according to IL-17/IFN-γ expression) of GM-CSF+ CD4+TCRαβ+ lymphocytes retrieved from draining lymph nodes of rats on the 7th day post-immunization (shown in D) is based upon fluorescence minus one controls: (A) minus GM-CSF, (B) minus IL-17 and (C) minus IFN-γ. CD4+TCRαβ+ lymphocytes were separated using magnetic-activated cell sorting (MACS) as described in Materials and Methods. This gating strategy was used in Fig 3. (TIF) [file pone.0166498.s006.tif]

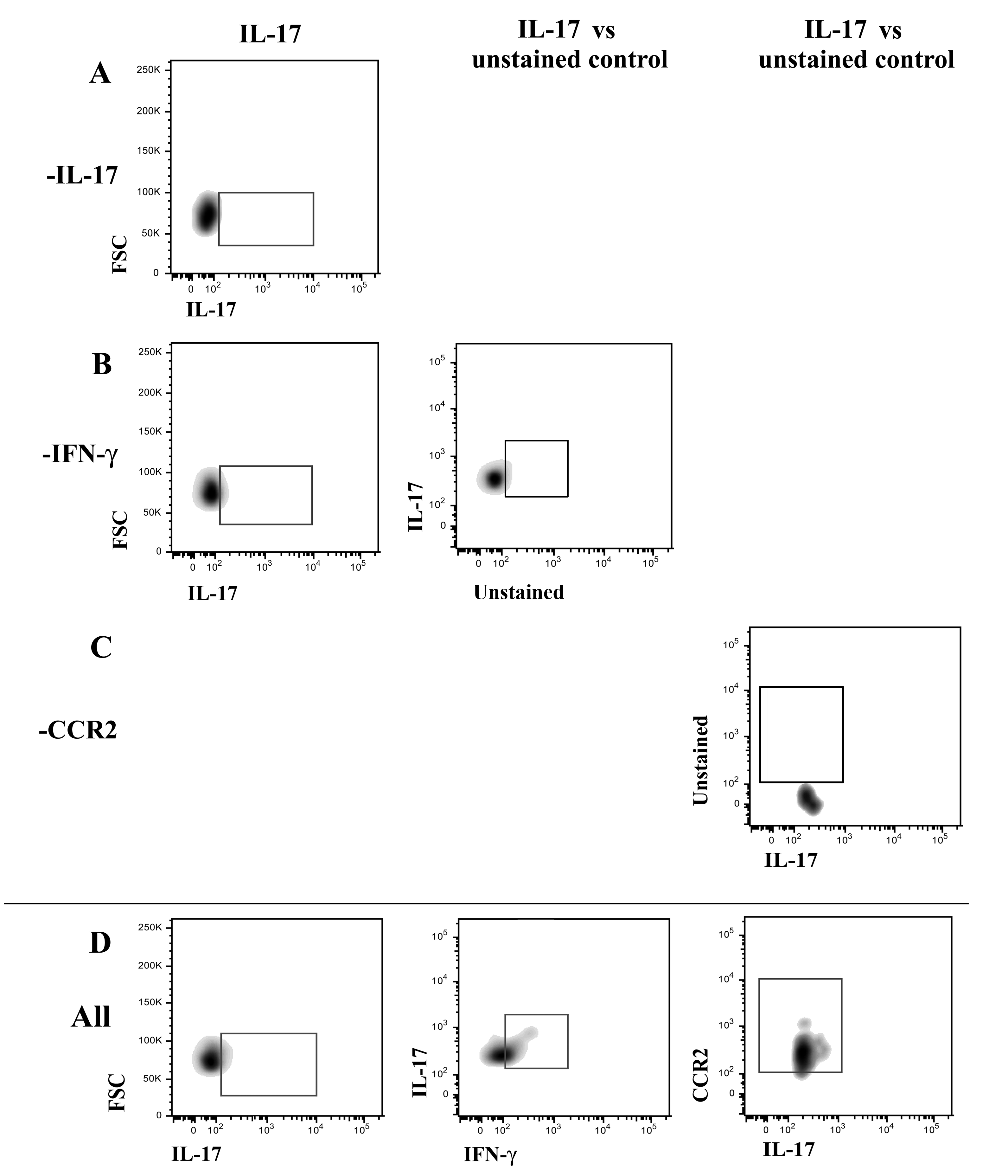

Supplement: S7 Fig — The gating strategy for CCR2-expressing IL-17+IFN-γ+ CD4+TCRαβ+ lymphocytes retrieved from draining lymph nodes of rats on the 7th day post-immunization (shown in D) is based upon fluorescence minus one controls: (A) minus IL-17, (B) minus IFN-γ and (C) minus CCR2. CD4+TCRαβ+ lymphocytes were separated using magnetic-activated cell sorting (MACS) as described in Materials and Methods. This gating strategy was used in Fig 5. (TIF) [file pone.0166498.s007.tif]

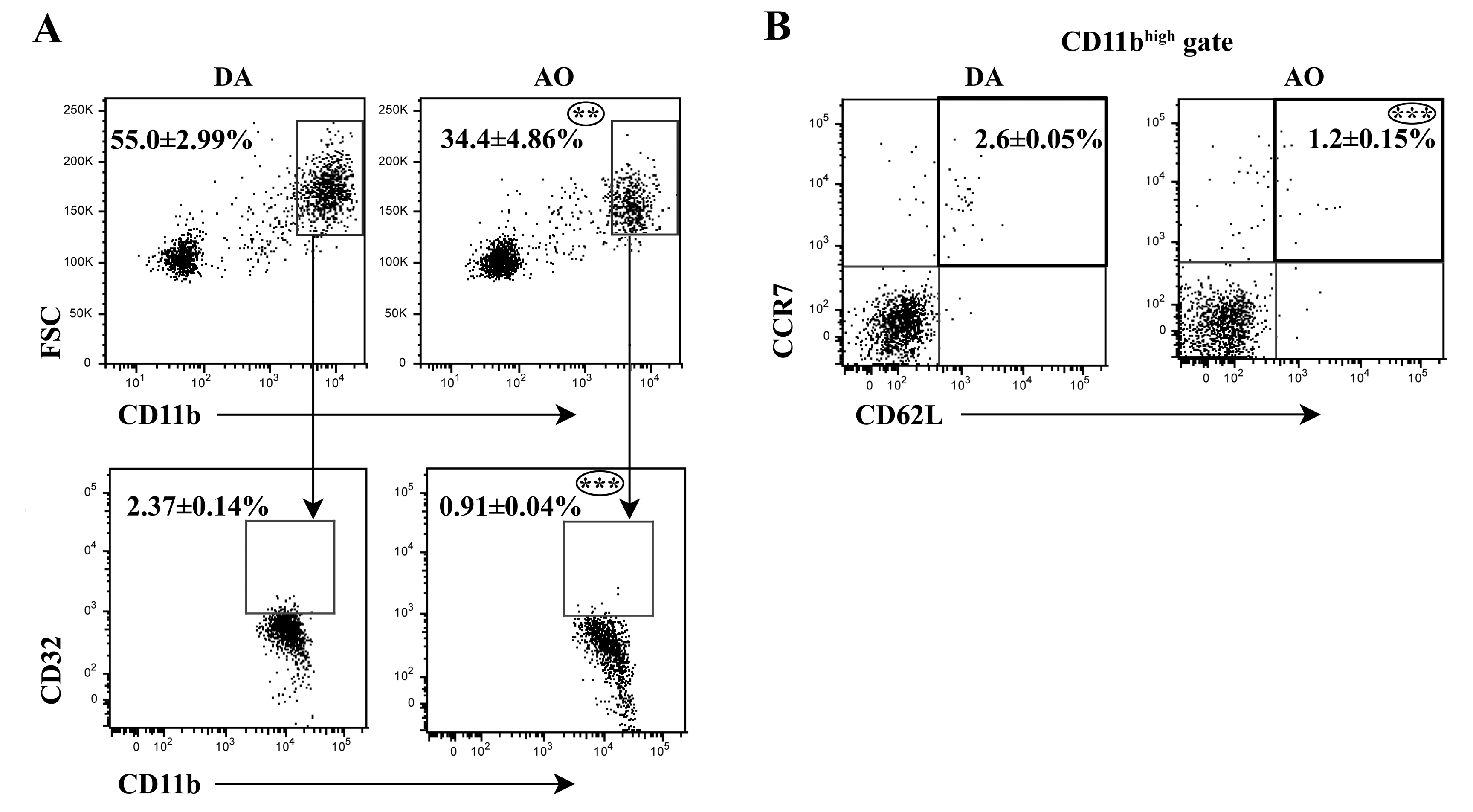

Supplement: S8 Fig — (A) Lower flow cytometry dot plots represent CD11b vs CD32 staining of CD11bhi peripheral blood (PB) cells retrieved from DA and AO rats on the 7th day post-immunization. The large CD11bhi PB cells are gated as shown in the upper flow cytometry dot plots. Numbers in the flow cytometry dot plots indicate the frequency of (upper) large CD11bhi cells within PB cells and (lower) CD32+ cells within CD11bhi cells. (B) Flow cytometry dot plots indicate CD62L vs CCR7 staining of CD11bhi PB cells of DA and AO rats. Numbers in the flow cytometry dot plots indicate the frequency of CCR7+CD62L+ cells within large CD11bhi PB cells gated as indicated (A). Data (mean ± SEM) are representative of two experiments (n = 6). ** p≤0.01; *** p≤0.001. (TIF) [file pone.0166498.s008.tif]

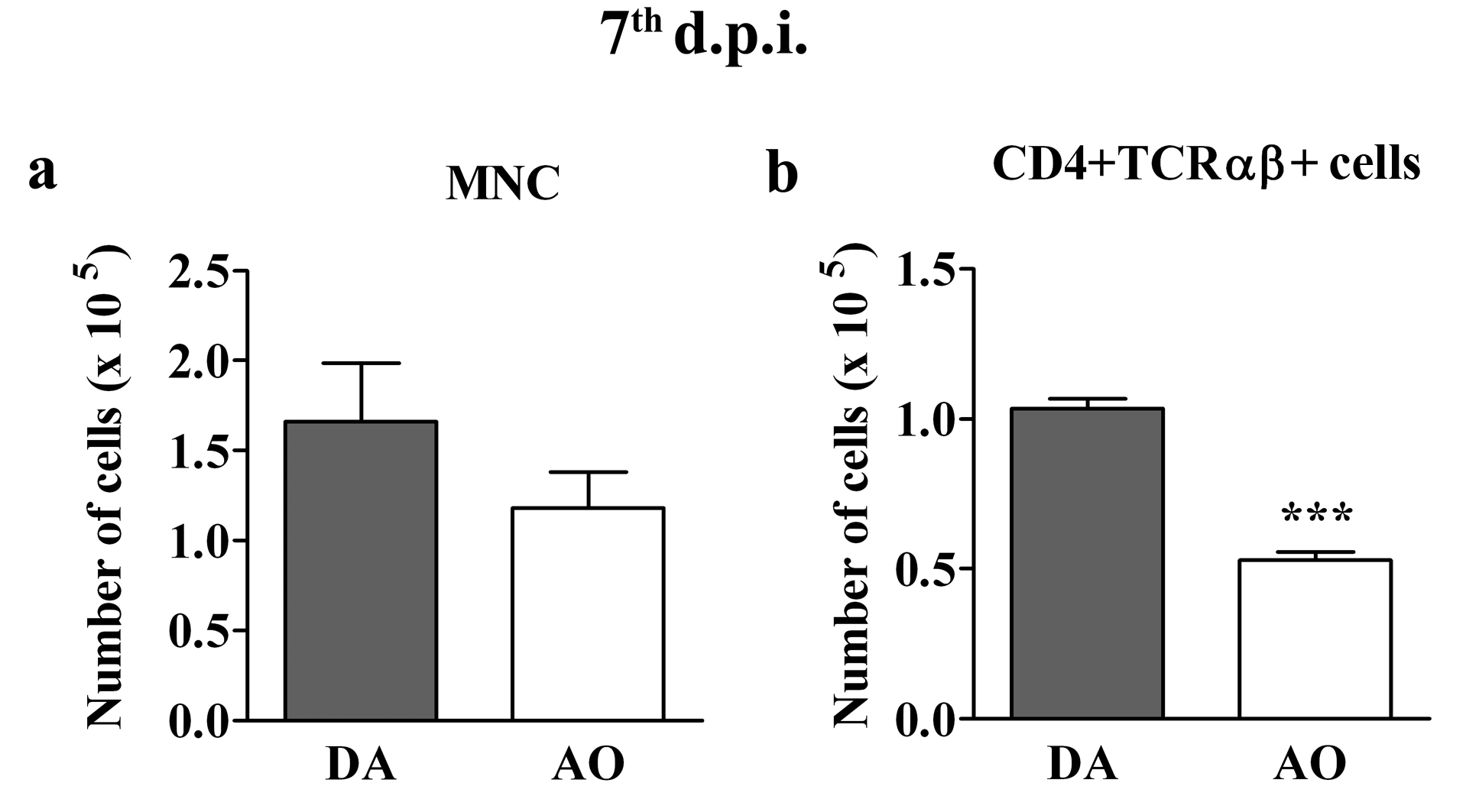

Supplement: S9 Fig — Bar graphs represent the number of (a) mononuclear cells (MNC) and (b) CD4+TCRαβ+ lymphocytes retrieved from spinal cord of DA and AO rats on the 7th day post-immunization (d.p.i.). Data (mean ± SEM) are representative of two experiments (n = 6). *** p≤0.001. (TIF) [file pone.0166498.s009.tif]

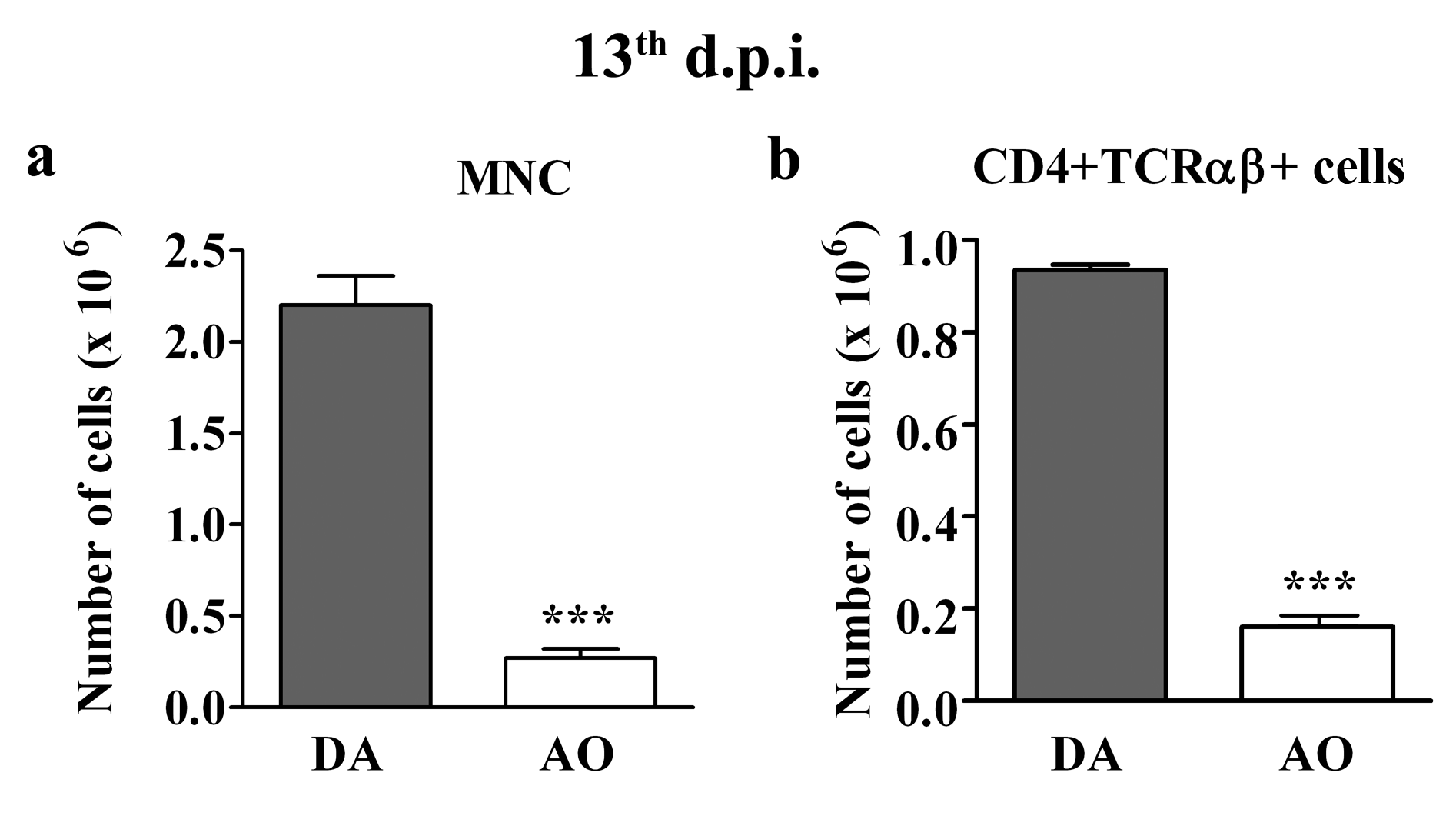

Supplement: S10 Fig — Bar graphs represent the number of (a) mononuclear cells (MNC) and (b) CD4+TCRαβ+ lymphocytes from spinal cord of DA and AO rat immunized for EAE on the 13th day post-immunization (d.p.i.). Data (mean ± SEM) are representative of two experiments (n = 6). *** p≤0.001. (TIF) [file pone.0166498.s010.tif]

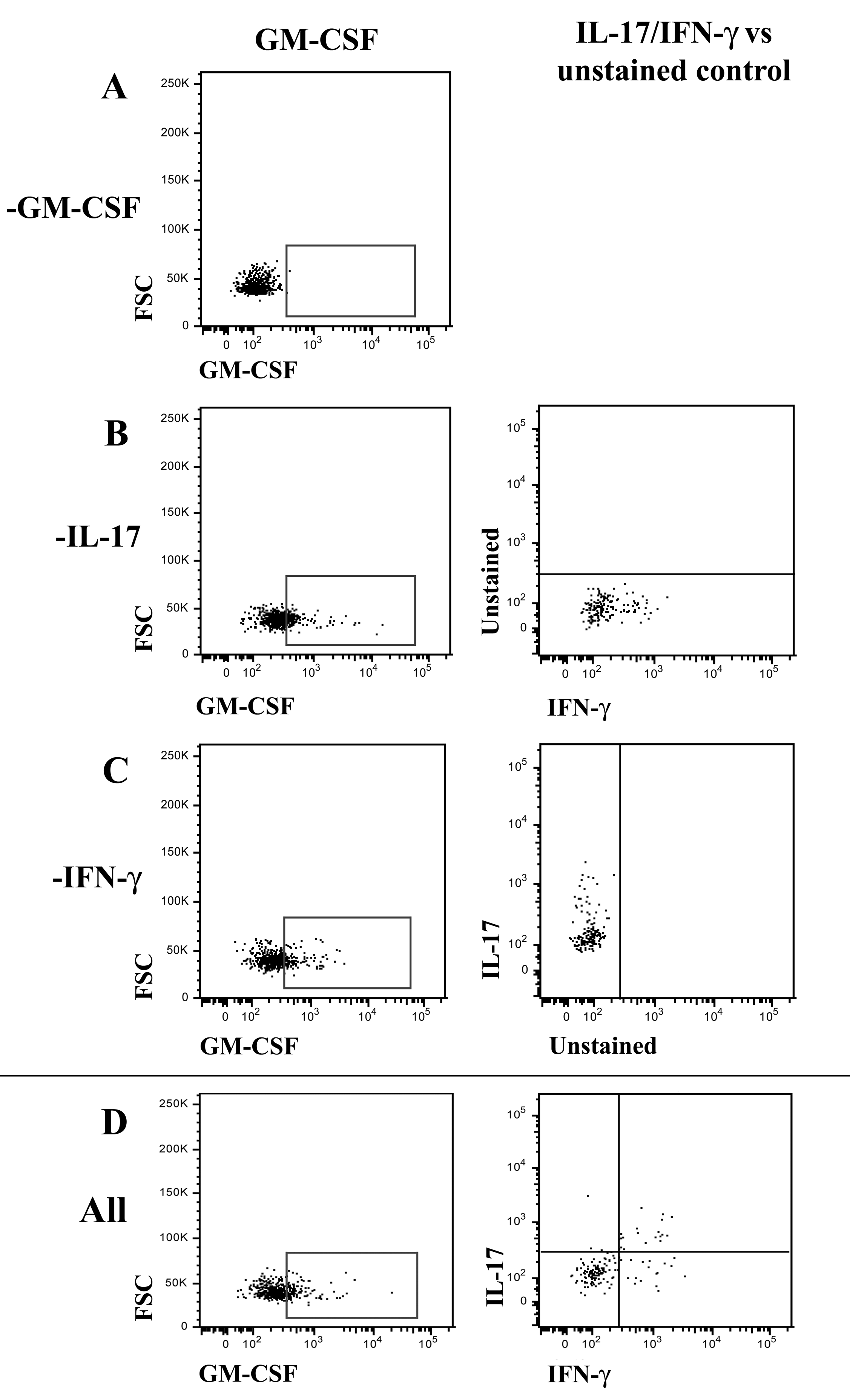

Supplement: S11 Fig — The gating strategy for distinct subsets (delineated according to IL-17/IFN-γ expression) of GM-CSF+ CD4+TCRαβ+ lymphocytes retrived from spinal cord of rats on the 13th day post-immunization (shown in D) is based upon fluorescence minus one controls: (A) minus GM-CSF, (B) minus IL-17 and (C) minus IFN-γ. CD4+TCRαβ+ lymphocytes were separated using magnetic-activated cell sorting (MACS) as described in Materials and Methods. This gating strategy was used in Fig 11. (TIF) [file pone.0166498.s011.tif]

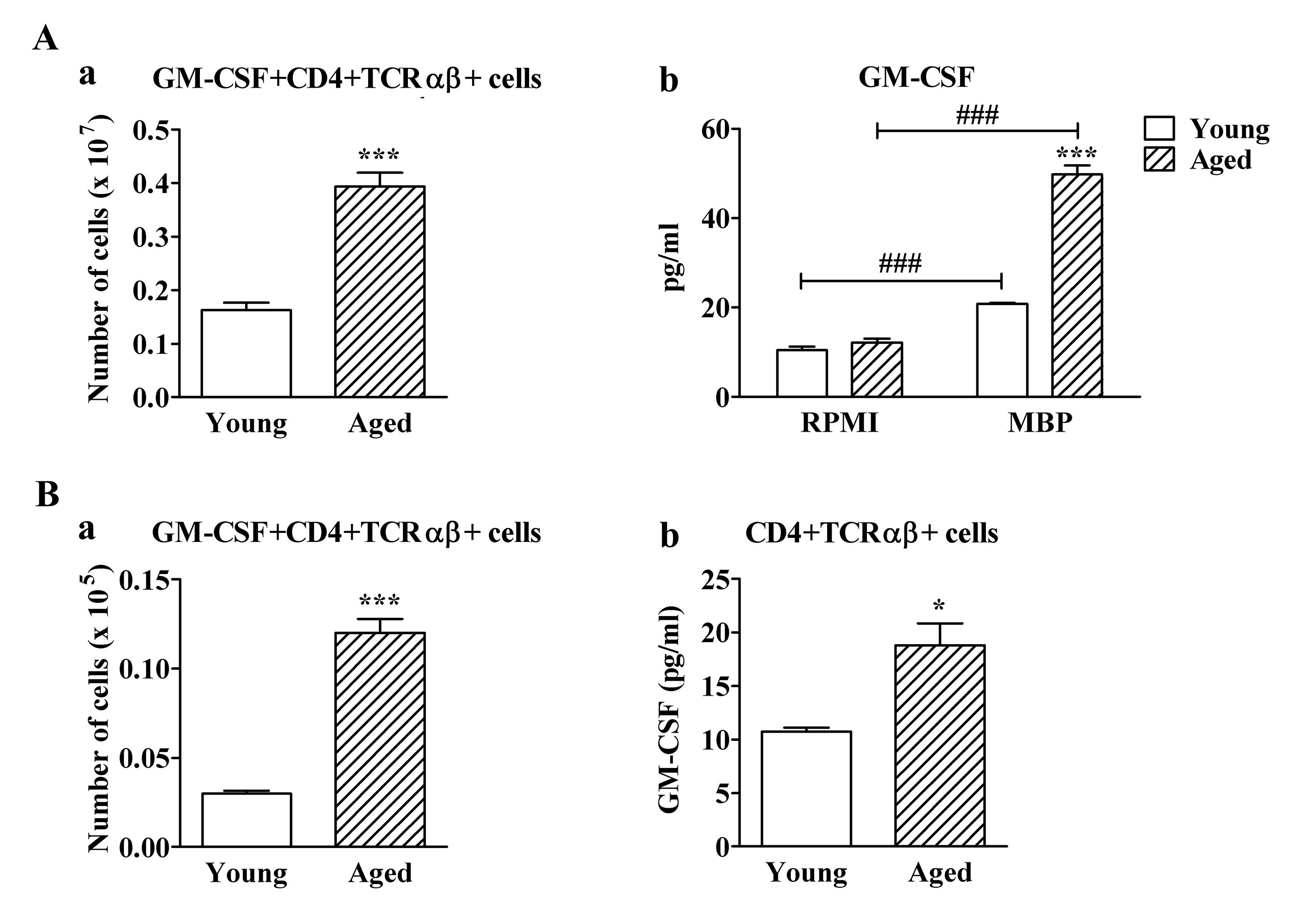

Supplement: S12 Fig — (A) Bar graphs represent (a) the number of GM-CSF+CD4+TCRαβ+ cells retrieved from draining lymph nodes (dLNs) of young and aged AO rats on the 7th day post-immunization (d.p.i.) and (b) concentration of GM-CSF in supernatants of dLN cells from young and aged AO rats cultured in RPMI alone or in the presence of MBP. (B) Bar graphs represent (a) the number of GM-CSF+CD4+TCRαβ+ cells retrieved from spinal cord (SC) of young and aged AO rats in the effector phase of the disease and (b) concentration of GM-CSF in supernatants of PMA/ionomycine stimulated CD4+TCRαβ+ lymphocytes retrieved from SC of young and aged AO rats. Data (mean ± SEM) are representative of two experiments (n = 9). * p≤0.05; *** p≤0.001; ### p≤0.001. * vs young AO rats; # vs RPMI. (TIF) [file pone.0166498.s012.tif]
