# Supplementary material for: GM-CSF-Producing Th Cells in Rats Sensitive and Resistant to Experimental Autoimmune Encephalomyelitis
Source: PLoS One. 2016 Nov 10;11(11):e0166498. doi: 10.1371/journal.pone.0166498 (PMC5104330; doi:10.1371/journal.pone.0166498)
Supplement: S1 Table — (DOC) [file pone.0166498.s013.doc]

**S1 Table**. Summary of mRNA targets and reference gene for RT-qPCR analysis.

|  | Symbol | Gene name | Accession No. **^a^** | Assay ID |
| --- | --- | --- | --- | --- |
| 1. | Il1b | Interleukin 1 beta | NM_031512.2 | Rn99999009_m1 |
| 2. | Il3 | Interleukin 3 | NM_031513.1 | Rn00580435_m1 |
| 3. | Il6 | Interleukin 6 | NM_012589.2 | Rn99999011_m1 |
| 4. | Il7 | Interleukin 7 | NM_013110.2 | Rn00681900_m1 |
| 5. | Il23a | Interleukin 23, alpha subunit p19 | NM_130410.2 | Rn00590334_g1 |
| 6. | Tgfb1 | Transforming growth factor, beta 1 | NM_021578.2 | Rn00572010_m1 |
| 7. | Csf2 | Colony stimulating factor 2 (granulocyte-macrophage) | NM_053852.1 | Rn01456850_m1 |
| 8. | Ccl2 | Chemokine (C-C motif) ligand 2 | NM_031530.1 | Rn00580555_m1 |
| 9. | Ccl20 | Chemokine (C-C motif) ligand 20 | NM_019233.1 | Rn01400118_g1 |
| 10. | Actb | Actin, beta | NM_031144.3 | Rn00667869_m1 |

**^a^** RefSeq: NCBI Reference Sequence Database.
